# Exploring Common Therapeutic Targets for Neurodegenerative Disorders Using Transcriptome Study

**DOI:** 10.3389/fgene.2021.639160

**Published:** 2021-03-19

**Authors:** S. Akila Parvathy Dharshini, Sherlyn Jemimah, Y. H. Taguchi, M. Michael Gromiha

**Affiliations:** ^1^Protein Bioinformatics Lab, Department of Biotechnology, Indian Institute of Technology Madras, Chennai, India; ^2^Department of Physics, Chuo University, Hachioji, Japan

**Keywords:** blood-brain barrier, Brodmann area-9, inflammatory response, energy dysfunction, transcription factor

## Abstract

Alzheimer’s disease (AD) and Parkinson’s disease (PD) are well-known neuronal degenerative disorders that share common pathological events. Approved medications alleviate symptoms but do not address the root cause of the disease. Energy dysfunction in the neuronal population leads to various pathological events and ultimately results in neuronal death. Identifying common therapeutic targets for these disorders may help in the drug discovery process. The Brodmann area 9 (BA9) region is affected in both the disease conditions and plays an essential role in cognitive, motor, and memory-related functions. Analyzing transcriptome data of BA9 provides deep insights related to common pathological pathways involved in AD and PD. In this work, we map the preprocessed BA9 fastq files generated by RNA-seq for disease and control samples with reference hg38 genomic assembly and identify common variants and differentially expressed genes (DEG). These variants are predominantly located in the 3′ UTR (non-promoter) region, affecting the conserved transcription factor (TF) binding motifs involved in the methylation and acetylation process. We have constructed BA9-specific functional interaction networks, which show the relationship between TFs and DEGs. Based on expression signature analysis, we propose that MAPK1, VEGFR1/FLT1, and FGFR1 are promising drug targets to restore blood-brain barrier functionality by reducing neuroinflammation and may save neurons.

## Introduction

Neurodegenerative disorders, the most debilitating form of progressive disorders, include Alzheimer’s disease (AD) and Parkinson’s disease (PD). The progression of neurodegeneration is associated with various symptoms based on the regions of neuronal cell death. These disorders share common pathological events such as selective vulnerability, insulin resistance, vascular dysfunction, protein aggregation, oxidative stress, calcium-induced glutamate toxicity, inflammatory response, aging, and ultimately neuronal death (Craft, 2007; Piehl and Olsson, 2009; Gan et al., 2018; Sweeney et al., 2018a; Muddapu et al., 2020). Genome-wide association studies, gene expression analysis from various tissue locations, and protein network studies reveal that genes related to mitochondria quality control, ubiquitin-mediated degradation, and endothelial tight junction genes are dysregulated in disease conditions (Guttula et al., 2012; Borrageiro et al., 2018; Lanke et al., 2018; Raj et al., 2018). Computational and experimental studies emphasize the deregulation of genes involved in energy metabolism and protein degradation processes that lead to neurodegeneration (Wang and Michaelis, 2010; Ciryam et al., 2016; Dharshini et al., 2019). Most microarray data and large-scale co-expression network studies suggest the importance of energy metabolism associated with neurodegeneration (Wang et al., 2007; Liang et al., 2008; Wang and Michaelis, 2010; Levine et al., 2013; Tiwari and Patel, 2014).

The neurovascular system plays an essential role in energy metabolism. Impairment in the blood-brain barrier (BBB) through various stress stimuli or cytotoxic inflammatory response affects glucose uptake and metabolism (Abbott, 2002; Freeman and Keller, 2012). Further, the neuronal population exhibits elevated energy demand for maintaining structural functional integrity and the regulation of homeostasis. In addition, it escalates the ROS (reactive oxygen species) response, depletion of antioxidants, and oxidative phosphorylation metabolism, which leads to neuronal stress and ultimately damaging the cell. These studies showed an imbalance in the energy reservoirs in the neuronal system, and it is necessary to revisit the pathways, which are essential in overcoming this energy imbalance. However, there is no systematic analysis of transcriptomic data on common therapeutic pathways for these diseases.

On the other hand, currently approved medications are meant to alleviate symptoms and slow down disease progression. There are no available therapeutics to save the surviving neuronal population. To determine potential therapeutic strategies, it is essential to identify the underlying cause of the disease.

At the early stages of the disease, hippocampal cornu ammonis 1 neurons (CA1) (Wilde et al., 1997) in Alzheimer’s and substantia nigra pars compacta neurons (SNc) in Parkinson’s disease (Damier et al., 1999) are more vulnerable to cell death compared to other neuronal populations. Various imaging techniques showed that these neurons are significantly reduced in patients (Karagulle et al., 2008; DeKosky and Scheff, 1990). These vulnerable neurons possess more synaptic terminals, i.e., dense arborization, and these complex structural phenomena may affect these neurons tremendously compared to other neuronal populations. This study aims to explore the common therapeutic target for neurodegenerative disorders. Since AD and PD share common pathological and symptomatic etiologies, the analysis of tissues affected by both diseases may help identify potential treatment targets. Brodmann area 9 (BA9) plays a key role in cognitive skills, executive memory, and motor behavior, and patients with AD and PD have demonstrated skills impairment, as mentioned earlier. In addition, reduced BA9 neuronal activity is observed in these patients. Since BA9 affects both diseases, we have selected this tissue for further analysis. Identifying variants, differentially expressed genes (DEGs), and tissue-specific network studies from high-throughput BA9 RNA-seq data provide clues for therapeutics.

We identified 167 common variants between AD and PD. These variants are also identified in Genome-Wide Association Studies (GWAS) and expression Quantitative Trait Loci studies (eQTL). These variants are predominantly located in the 3′ UTR region, creating or disrupting the conserved regulatory binding motifs and affecting the transcription factor (TF) binding sites located explicitly in the non-promoter region (Albert and Kruglyak, 2015). Several variants affect the conserved TF motif associated with histone acetylation and demethylation, thus impairing downstream gene expression (McGuire et al., 2019). From tissue-specific network analysis, we identified TFs, which activate or repress the differentially expressed (DEG) genes in BA9. Tissue-specific functional module analysis revealed that endothelial and vascular smooth muscle cell pathways are dysregulated. These pathways are vital for preserving blood-brain barrier (BBB) stability and cerebral blood pressure regulation (Zenaro et al., 2017). From this study, we propose that mitogen-activated protein kinase-1 (MAPK1), vascular endothelial growth factor receptor-1 (VEGFR1), and fibroblast growth factor receptor-1 (FGFR1) serve as promising drug targets, which may help to preserve vascular endothelial pathways and reduce chronic inflammation. Further exploring these targets may restore BBB integrity and save neurons from the energy crisis and associated neuronal death.

## Materials and Methods

### Variant Calling and Predicting the Effect of Variants

The BA9 RNA-seq data were retrieved from a sequence retrieval archive (SRA) for AD, PD, and control samples (Supplementary Table S1), which included nine AD, 28 PD, and 52 age-matched control samples (Dumitriu et al., 2016; Scheckel et al., 2016). The data were retrieved from post-mortem samples after death (2–6 h). The average RNA integrity value for the sample was above 8 which denotes the stability of the mRNA.

The RNA-seq raw reads were preprocessed using the NGSQC toolkit (Wang et al., 2015), and reads with a PHRED score less than 20 were discarded. The index-specific Illumina adapters were removed using the Trim Galore tool (Krueger, 2015). The preprocessed reads were subjected to spliced alignment using hg38 genomic assembly and the STAR2.6 aligner (Dobin et al., 2013). After spliced alignment, the samples’ alignment rates were above 80% (uniquely mapped reads). We eliminated duplicates using PICARD to reduce the erroneous read depth during variant calling. We recalibrated the base quality score near the variant site using the GATK4 (McKenna et al., 2010) recalibrating module. The Haplotype caller was used to identify the variants. We imposed hard filters such as PHRED quality score above 30 (denotes the variant base as 99.99% accurate) and supporting read depth for variant ≥10. We categorized the variants exclusively present in the disease population but not included in the control subjects. We compared the variants with the Genotype-Tissue Expression (GTEx) consortium (Carithers and Moore, 2015), which helps filter out variants located in healthy brain tissues. We compared the identified variants with an xQTL study and discarded the matched hits for considering the aging effect. The xQTL study (Ng et al., 2017) mainly included samples from the healthy aged subjects from BA9. The shortlisted variants were compared with various eQTL and GWAS of AD and PD (Supplementary Table S2; Pankratz et al., 2012; Lambert et al., 2013; Greene et al., 2015; Deming et al., 2017; Jun et al., 2017). We selected common variants present in AD and PD pathogenesis for further analysis. Most of the variants were located in the 3′ UTR region and we predicted the effect of variants on transcription factor binding using Haploreg, SNP2TFBS, and GWAS4D (Ward and Kellis, 2012; Kumar et al., 2017; Huang et al., 2018). The effect of non-coding variants was predicted using the *in silico* tools CADD, DeepSea, Eigen, GWAVA, Funseq2, FATHMM, and REMM (Dayem Ullah et al., 2018).

3′ UTRs are involved in numerous regulatory processes, including transcript cleavage, RNA binding protein, stability and polyadenylation, translation, and mRNA localization. These 3′ UTRs contain some of the most conserved regulatory elements within the mammalian genome and they serve as binding sites for numerous regulatory RNA binding proteins and microRNAs. The effect of variants on miRNA binding was predicted using miRbase and vista (Dayem Ullah et al., 2018), variants on RNA binding proteins were predicted using the RBP-var web-based tool (Mao et al., 2016), and miRNA binding targets were predicted using the miRDB tool (Chen and Wang, 2020).

### Computing the Variant Nucleotide Frequency Based on Genomic Location

We computed the propensity of nucleotides for the identified variants based on their genomic locations [control/AD/PD(BA9)/GWAS(AD/PD)] for determining the preferences of nucleotide changes in AD/PD samples. Supplementary Table S3 shows the frequency of nucleotides based on their genomic location for the reference human genome. From Supplementary Table S3, we observed that the occurrence of nucleotide base T was higher in the intronic and UTR3 regions. The occurrence of C and G was higher compared to other bases in the upstream and UTR5 regions.

(1) $${\text{P}}_{\text{N}}={\text{f}}_{\text{N}}/\Sigma_{A,T,C,G}\left\{N=A,T,C,G\right\}$$

(2) $${\text{P}}_{i\to j}={\text{f}}_{i\to j}//\Sigma_{A,T,C,G}\left\{i,j=A,T,C,G\right\}$$

(3) $$\text{P}\left[\left(i\to j\right);i\right]={\text{P}}_{i\to j}/{\text{P}}_{\text{i}}$$

We calculated the propensity matrix for the variants using Eqs (2) and (3). For example propensity of i→j (A→T) change in a given sample (control, AD/PD, GWAS) is calculated using the occurrence of i→j change in the specified genomic region (intronic/upstream/UTR3/UTR5/exonic) divided by the occurrence of i in the human genome located in the same genomic region.

### Differential Gene Expression

The preprocessed RNA-seq reads were aligned with the hg38 human transcriptome (ensemble genomic build) using the Salmon quantification (Patro et al., 2017). The counts were normalized using transcript length and library size. We calculated the gene abundance using tximport. We performed differential gene expression using DEseq2 (Love et al., 2014), and rigorous statistical testing to filter the gene expression level (Benjamini–Hochberg Q value < 0.05, minimum fold change | log_2_ FC| > 1). We only selected genes that were upregulated (or) downregulated in both disease conditions from the BA9 RNA-seq profile data. We compared the gene expression fold change with various RNA-seq and microarray datasets available in the literature [single cell (Lau et al., 2020) and other tissue RNA-seq data (Donega et al., 2019; Simchovitz et al., 2020; Srinivasan et al., 2020)]. The identified differentially expressed genes (BA9) were compared with the GTEx consortium to understand the expression pattern in other normal tissues (skin, heart, bladder, kidney, spinal cord, blood, BA9, adipose, small intestine, lungs, and pancreas) that are not affected by these diseases. The median transcript per million counts (TPM) for various normal tissues was compared to BA9 [control and disease (AD/PD)].

### Tissue-Specific Functional Interaction/Co-expression Network and Enrichment Analysis

We built a BA9-specific functional interaction network between the variant associated genes, transcription factors (TFs), and differentially expressed genes (DEGs) using Reactome FI, the KEGG parser pathway tuning module, and HIPPIE web tools (Wu et al., 2014; Alanis-Lobato et al., 2017). This study helps to understand the functional relationship between TFs and DEGs. We constructed the co-expression network using the information available in the HumanBase and TCSBN databases (Greene et al., 2015; Lee et al., 2018). This network aids in interpreting genes, which are co-expressed together in BA9. We performed network analysis such as degree and centrality measures to identify the network hubs. The identified DEGs and TFs were subjected to tissue-specific functional module analysis using HumanBase. The functional significance of these gene sets were elucidated by enrichment analysis.

### Expression Signature/Perturbagen Analysis

The Connectivity map (cMAP) and LINCS web-based tools include transcriptional expression data accumulated from various perturbations (genetic, small molecule), which help to identify drug molecules based on disease-specific upregulated gene expression profiles (Lamb et al., 2006; Subramanian et al., 2017; Stathias et al., 2020).

1. We submitted the list of upregulated genes to these portals and obtained a set of drug molecules along with their respective targets. The scores ranged from -100 to + 100, which denotes that a given drug molecule is positively or negatively connected with a given target. These scores were derived from cMAP. For this analysis, we selected drug molecules which scored more than 85.
2. We also overlaid drug molecules from various repositories such as chEMBL, DrugBank, and PubChem to the functional interaction network using the Reactome FI cytoscape app.
3. We identified common drug molecules and their respective targets using both methods (1) and (2). We shortlisted the inhibitors and carefully reviewed the literature to understand the functionality of selected targets for further explorative studies. We have represented the workflow in Supplementary Figure S1.

## Results

### Variant Analysis of BA9 Samples Obtained From AD and PD

We analyzed the BA9 samples of AD and PD and identified 167 variants present in both AD and PD samples, which were not present in the control population. The identified variants from the current study were compared with various GWAS data (AD/PD) to understand whether the variant was already reported for the disease or not. GWAS provides information on the variants predominantly present in the disease population, but the effect of those variants on transcription factor binding, expression, and miRNA binding are not explored. Hence we used *in silico* tools to predict the impact of the variants. Supplementary Figure S2A shows that 128 variants residing in non-coding regions may play an essential role in gene regulation (Siepel et al., 2005; Araujo et al., 2012). Most of the variants were found in non-coding regions in both control and disease samples. But in the disease sample, the number of 3′ UTR variants was higher compared to other regions. GWAS studies showed that most of the disease-associated variants were located in the regulatory region and in the UTR regions compared to coding segments (Ma et al., 2015; Steri et al., 2018). We also calculated the percentage of variants in each genomic location [control, AD/PD(BA9), and GWAS variants (AD/PD)], and the results are shown in Supplementary Figure S2B. We observed that intronic variants were higher than the coding segments due to the high percentage of the intronic region in the human genome compared to the coding region.

We also computed the nucleotide frequency for the identified variants based on their genomic locations [control/AD/PD(BA9)/GWAS(AD/PD)] for determining the preferences of nucleotide changes in AD/PD samples. In the control dataset, A- > G, T- > C variants were high in the intronic region. In disease samples, the frequency of G- > A, C- > T was high both in the current study and in GWAS studies (Supplementary Table S2). This shows that epitranscriptomic modification plays an essential role in the disease mechanism (Angelova et al., 2018). Detailed information about the nucleotide frequency is represented in Supplementary Table S4.

Mostly identified variants were located in the non-promoter region. Mapping regulatory features such as enhancers and conserved transcription factor binding motifs to these variants may help to predict the effect. Albert and Kruglyak (2015) reported that the 3′ UTR variant found in the CELSR2 gene disrupts the cis-regulatory binding motif of C/EBP transcription factor (TF), and subsequent eQTL studies have shown that this variant affects the gene expression of SORT1 which is 40kb away from the variant site and is involved in myocardial infarction. We predicted the variant effects using various *in silico* tools (described in the section “Materials and Methods”). Of the 167 identified variants, 103 affected the binding affinity of 47 known transcription factors, which are already implicated in AD and PD. Microarray studies reported in the literature showed that these TFs are dysregulated in both disease conditions (Blalock et al., 2004; Lesnick et al., 2007; Zheng et al., 2010; Narayanan et al., 2014; Wang et al., 2016; Berchtold et al., 2019; Patel et al., 2019; Piras et al., 2019). Table 1 lists some of the crucial variants affecting the conserved regulatory motifs of the transcription factors involved in both diseases. Detailed information about the identified variants and their predicted effects are represented in Supplementary Table S5.

**TABLE 1:** Common variants and their effect on regulatory elements.

**Chromosome**	**Position**	**Reference allele**	**Alternative allele**	**Genomic location**	**Gene name**	**SNP ID**	**Regulatory element (TF)**	**AD/PD TF expression (fold change)**
11	72756232	C	T	Intronic	STARD10	rs148235301	CREBBP	Up (2.7, 1.4)
4	959671	G	A	UTR3	DGKQ	rs75067698	**DNMT1**	**Up (5.4, 3.8)**
8	101689173	G	A	UTR3	NCALD	rs113375628		
12	57095374	C	T	UTR3	NAB2	rs3024983		
18	48859991	G	T	UTR3	CTIF	rs141179242		
19	1376842	C	T	UTR3	MUM1	rs139291622	**HDAC1**	**Down (−4.7, −3.5)**
5	6668802	G	A	UTR3	SRD5A1	rs1042150	**KAT2B**	**Up (6.8, 2.7)**
7	19696657	T	C	UTR3	TWISTNB	rs17354985	MEF2A	Up (2.7, 1.4)
17	3636541	T	C	UTR5	CTNS	rs111977802	REST	Down (−2.7, −4)
12	84861228	T	C	UTR3	SLC6A15	rs143168309	RXRA	Down (−4.2, −5.5)
7	76273420	G	A	Intronic	SRRM3	rs77373389	ZBTB7A	Up (8.7, 3.7)
14	77028141	G	C	UTR5	IRF2BPL	rs76980172		
15	90947604	C	T	Intronic	UNC45A	rs144002184		
10	79386308	T	C	UTR3	ZCCHC24	rs147555076	BCL2	Up (3.5, 2.2)

*The highlighted genes are involved in histone acetylation and methylation.*

DNA methylation plays a crucial role in epigenetic regulation and gene expression. The 3′ UTR region is enriched with methylation sites and is positively correlated with gene expression profile (McGuire et al., 2019). Studies showed that (Wang et al., 2019) 3′ UTR variants affect the methylation pattern as well as gene expression. We observed that the identified variants, which mainly affected the regulatory elements, were involved in demethylation (DNMT1), histone acetylation (KAT2B), and deacetylation (HDAC1), and these genes are dysregulated in AD and PD. Table 1 shows that the STARD10 [rs14823530 (C/T)] variant disrupts the binding of CREBBP TF and regulates other transcription factors involved in synaptogenesis through histone acetylation (Valor et al., 2013). CREBBP is upregulated in both diseases. Variants such as DGKQ [rs75067698 (G/A)], NCALD [rs113375628 (G/A)], NAB2 [rs3024983(C/T)], and CTIF [rs141179242(G/T)] affect DNMT1 binding. DNMT1 is involved in DNA methylation and thereby controls gene expression and is upregulated in AD and PD. The SRD5A1 [rs1042150 (G/A)] variant disrupts KAT2B TF binding and is involved in transcription activation by transferring the acetyl group. KAT2B is upregulated in AD and PD. The MUM1 [rs139291622 (C/T)] variant disrupts HDAC1 binding, and this TF is implicated in transcriptional repression by deacetylation, which is downregulated in AD and PD (CoppedÃ, 2014). The TWISTNB [rs17354985 (T/C)] variant impairs the MEF2A TF binding. This TF negatively regulates mitochondrial function which dampens ATP functionality and is upregulated in both diseases. The CTNS [rs111977802 (C/T)] variant disrupts the binding of REST which participate in neurogenesis. REST is downregulated in both diseases. SRRM3 [rs77373389(G/A)], IRF2BPL [rs76980172 (G/C)], and UNC45A [rs144002184 (C/T)] variants disrupt ZBTB7A TF binding. This TF acts as a transcriptional repressor and is upregulated in AD and PD. This analysis revealed an imbalance in the transcriptional network that leads to aberrant activation or inactivation of downstream genes. Additionally, the ZCCHC24 [rs147555076(T/C)] variant disrupts Bcl2 binding, is involved in apoptosis regulation, and is upregulated in these diseases. Furthermore, we identified that the SZRD1 [rs138678090 (C/T)] and YWHAB [rs188983062 (C/G)] variants affect hsa-miR-301a-3p and hsa-miR-212-3p miRNA binding. These miRNAs are downregulated in both diseases, as reported in the literature using miRNA signature studies (Kumar and Reddy, 2016; Pallarès-Albanell et al., 2019). 3′ UTR variants such as rs72984526 (CAPZA1), rs149358308 (PBX1), rs1042150 (SRD5A1), and rs41305489 (CENPP) affect the RNA structure (riboSnitch) along with the RBP binding motif, which in turn affects miRNA binding. Details are given in Supplementary Table S5. In summary, we observed the effect of variants at multiple hierarchies such as 27 TFs, two miRNA, and 30 RBP binding motifs which were altered by 3′ UTR variants. We represent this information in Supplementary Figure S3 along with the tools used to predict the effect of variants.

### Differentially Expressed Genes in AD and PD

We identified differentially expressed genes with similar gene expression profiles in both AD and PD. We observed that 100 and 98 genes were up and downregulated in AD and PD pathogenesis, respectively. The details of count tables along with the volcano plot are given in Supplementary Table S6 and Supplementary Figure S4.

#### Comparison of BA9 Gene Expression (Disease) With GTEx Normal Tissue Expression

The upregulated and downregulated gene expression profiles of the BA9 disease samples were compared with the GTEx consortium to understand the expression pattern in other normal tissues that are not affected by these diseases. The details are provided in Supplementary Figures S5, S6. The TPM value was high for the disease BA9 tissue for upregulated genes and low for downregulated genes. The pattern of gene expression in the disease sample differed from that of other control tissues.

#### Comparison of BA9 Gene Expression (Disease) With Other RNA-Seq Data (AD/PD)

Due to the unavailability of BA9 RNA-seq data in both diseases, we compared expression profiles with recently published RNA-seq data from various other tissues. Details are provided in Supplementary Table S7.

**FIGURE 1:**
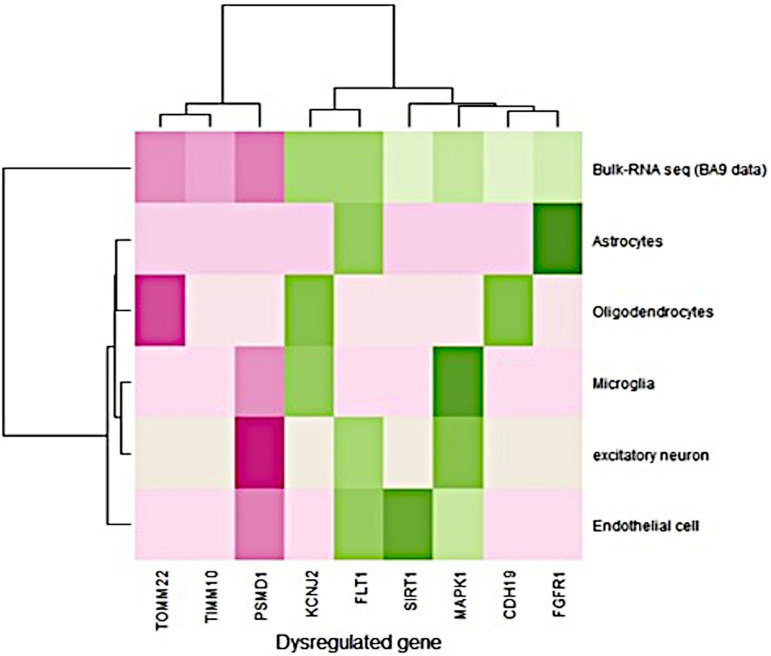
Comparison of gene expression profiles between bulk and single nuclei RNA-seq (disease/BA9).

The single-cell RNA-seq data obtained from the prefrontal cortex (BA9) were available for Alzheimer’s disease. It consisted of 80,660 cells from six different cell types derived from 12 AD disease samples (Lau et al., 2020). The expression pattern (fold change) of single nucleus RNA-seq data was compared to the bulk RNA-seq data expression pattern (BA9-current study). Bulk RNA-seq data showed a similar expression pattern with single-cell RNA-seq data (Figure 1). FLT1 gene expression was high in endothelial, astrocytes, and excitatory neurons, and bulk RNA-seq also captured this gene as upregulated in disease conditions. PSMD1 was downregulated in endothelial cells, excitatory neurons, and microglia and bulk RNA-seq data showed that this gene was downregulated in disease conditions. Detailed information about the fold change in bulk and single nuclei RNAseq data (BA9) are given in Supplementary Table S7. Most of the gene expression patterns matched the single-cell RNA seq data.

#### Biological Classification of Differentially Expressed Genes

**FIGURE 2:**
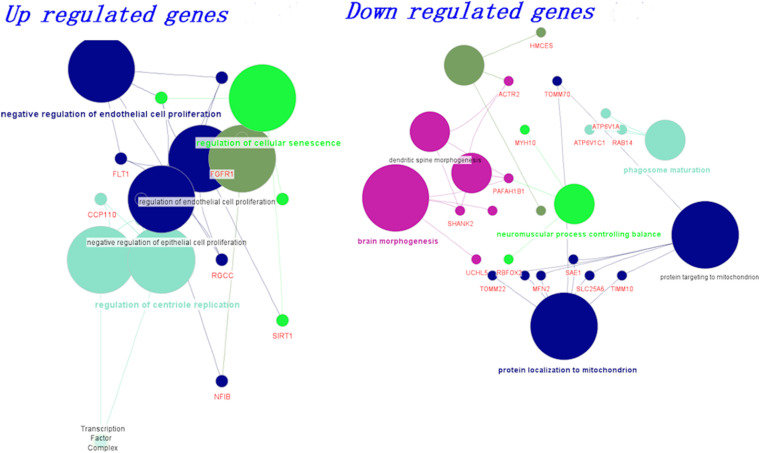
Biological classification and functional enrichment modules for upregulated and downregulated genes in AD and PD pathogenesis. Modules are shown in different colors and the size of the node denotes the number of genes.

We grouped the differentially expressed genes based on up and downregulation. Using the clueGo cytoscape app, we classified genes based on their biological functions. The functional enrichment modules for upregulated and downregulated genes in AD and PD pathogenesis are shown in Figure 2. Furthermore, genes related to negative regulation of endothelial function and cellular senescence were upregulated, while genes involved in mitochondrial function and neuromuscular processes were downregulated. The list of differential gene expression is given in Supplementary Table S6. Upregulation of the FLT1 and FGFR1 genes may affect the blood-brain barrier (Mahoney et al., 2019; Chen et al., 2020; Lau et al., 2020). Additionally, cellular senescence is provoked by various factors such as mitochondrial dysfunction, oxidative stress, inflammation, and protein mishandling. The upregulation of senescence-related genes (BCL6, SIRT1) may lead to the deterioration of functional features.

On the other hand, mitochondrial localization (MFN2, TIMM10, TOMM22) and neuromuscular function (MYH10, RBFOX2, PAFAH1B1)-related genes were downregulated. We observed that the dysregulation of genes related to endothelial function influence BBB damage, which leads to impairment in vascular dynamics. Additionally, mitochondrial function-related genes were dysregulated and led to inadequate energy resources (Bélanger et al., 2011; Freeman and Keller, 2012; Sweeney et al., 2018a). All these events led to oxidative stress, which ultimately influences cellular senescence (Sweeney et al., 2018a). In addition, we also generated a boxplot based on the normalized count values obtained for these genes using control, AD, and PD datasets, and the data are shown in Supplementary Figure S7.

### Effect of Variant, miRNA Binding, and Downstream Expression

**FIGURE 3:**
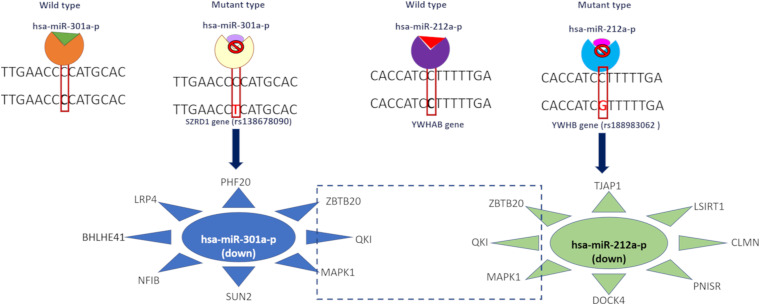
Effect of 3′ UTR variants in miRNA binding and downstream gene expression.

We observed that the 3′ UTR gene variants SZRD1 and YWHAB affected the regulatory binding motifs of hsa-miR-301a-P and hsa-miR-212a-p binding (Figure 3). These miRNAs were downregulated in both disease conditions. We predicted potential miRNA targets and observed that these targets were upregulated in both disease conditions. hsa-miR-301a-p miRNA targets the genes SUN2, NFIB, LRP4, and PHF20 whereas hsa-miR-212a-p targets DOCK4, PNISR, CLMN, LSIRT1, and TJAP1. All these genes were upregulated in both disease conditions. Both of these miRNA target the following genes MAPK1, QKI, and ZBTB20 and these genes are upregulated in AD and PD (Supplementary Table S6).

### Tissue-Specific Functional Interaction and Co-expression Networks

We identified common variants, which may create or disrupt the conserved regulatory binding motifs of TFs involved in AD and PD. These TFs may activate or inhibit various downstream genes. To understand the functional relationship between TFs and DEGs, we built a tissue-specific interaction network. We also identified fewer overlaps between these classes (TF, DEG, and variants). The below mentioned genes overlapped in either of two groups: TF/DEG TCF12, variant/DG FLT1, and variant/TF TAL1.

**FIGURE 4:**
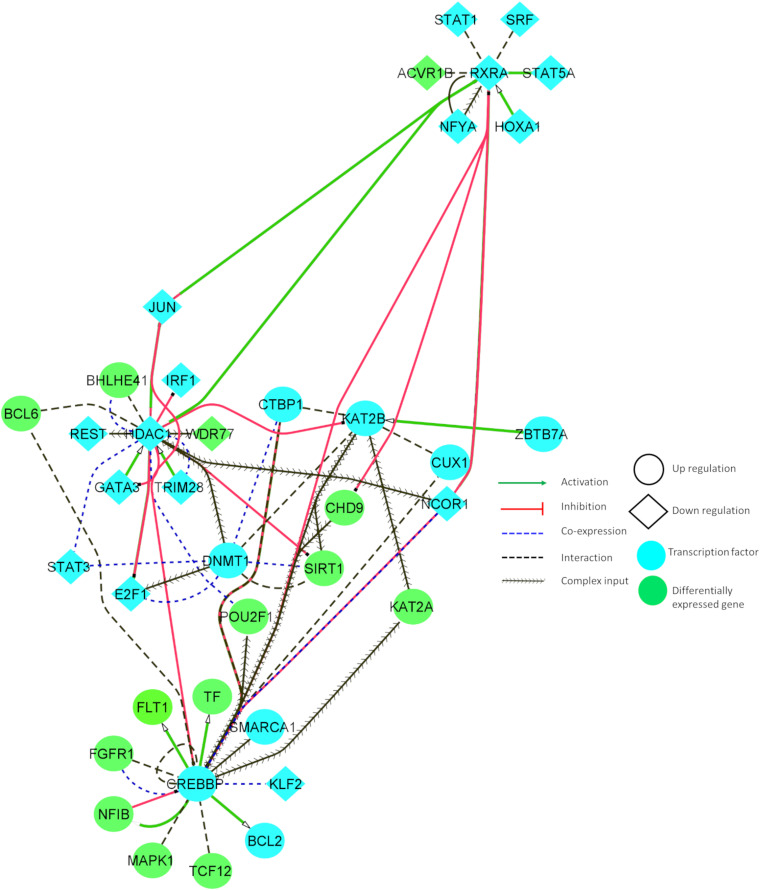
Tissue-specific (BA9) functional interaction network between transcription factors and differentially expressed genes involved in AD and PD.

Figure 4 illustrates the interaction between some of the essential TFs and DEGs. These TFs showed increased connectivity (degree > 5). The relationship between the identified DEGs and TFs can reveal important insights into neuronal cell survival. Figure 4 denotes that CHD9 activates CREBBP, and both TFs are upregulated in AD and PD. NCOR1 and JUN inhibit CREBBP, and these TFs are downregulated and co-expressed in BA9. KAT2B and FGFR1 interact with CREBBP, and these genes are co-exprssed and upregulated in AD and PD. CREBBP activates FLT1, CTBP1, BCL2, NFIB, BCL6, PBX1, and CUX1. All these genes are upregulated in both diseases. CREBBP TF binding is affected by the intronic variant (Table 1). CHD9, HDAC1, PBX1, JUN, CTBP1, CREBBP, and KAT2B genes are involved in NOTCH signaling, which is essential for endothelial cell migration, axonal sprouting, and neuronal survival (Lasky and Wu, 2005).

JUN, TRIM28, and E2F1 activate HDAC1, and all these genes are downregulated in disease conditions. HDAC1 inhibits E2F1, IRF1, JUN, and GATA3, and these genes are downregulated in AD and PD. HDAC1 interacts with BHLHE41, DNMT1, REST, and BCL6, and is co-expressed in BA9. DNMT1 TF binding is affected by UTR variants (Table 1). E2F1, JUN, and BCL6 are involved in cellular senescence and autophagy. JUN, DNMT1, and REST genes are implicated in AKT cell survival signaling, which shows that genes participate in autophagy, and cell survival signaling is dysregulated in both of the disease conditions.

KAT2B is activated by ZBTB7A and upregulated in disease conditions. KAT2B inhibits HDAC1 and interacts with CREBBP, DNMT1, KLF2, KAT2A, SIRT1, CTBP1, E2F1, and JUN, and is co-expressed in BA9. KAT2B TF binding is affected by UTR variants (Table 1). Major constituents of these gene sets are involved in NOTCH signaling and oxidative stress related senescence. IRF1 and FGFR1 activate MAPK1, and these genes are upregulated in the disease conditions. MAPK1 regulates the expression of YWHAB, RXRA, STAT3, FGFR1, and BCL2 genes. These genes participate in synaptic transmission and are dysregulated in disease conditions.

Downregulated RXRA activates STAT5A, JUN, and NCOR1, and these genes are downregulated in these disorders. RXRA inhibits CHD9 and CREBBP. RXRA TF binding is affected by UTR variants (Table 1). Dysregulation of these genes resulted in aberrant long term potentiation and survival signaling pathways. From this study, we found an inherent relationship between TFs and DEGs in disease conditions which may involve various crucial signaling pathways, including the regulation of endothelial cells, oxidative stress response, and cell survival pathways. Detailed information about the functional interaction and co-expression of other genes are provided in Supplementary Table S8.

### Functional Module Network Analysis

**FIGURE 5:**
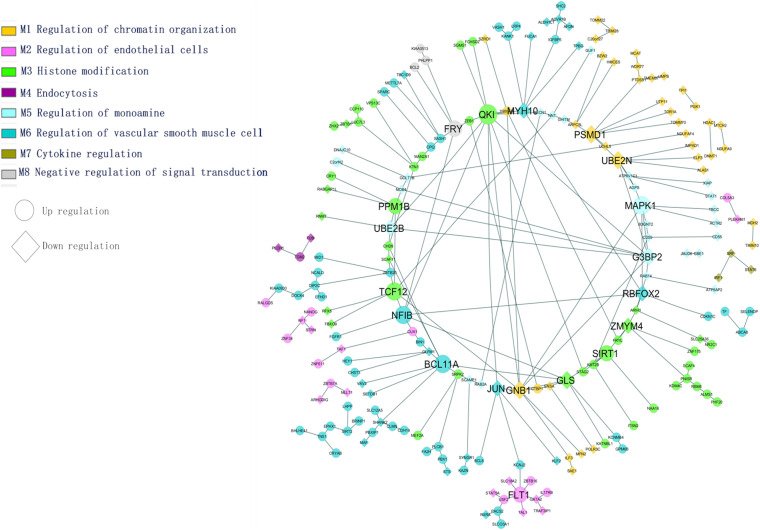
Tissue-specific (BA9) enrichment functional module network (transcription factors and differentially expressed genes).

The most common identified differentially expressed genes and transcription factors were subjected to tissue-specific functional enrichment analysis. Modules were denoted as enriched biological functions obtained from the tissue-specific community network from the list of DEGs/TFs. Figure 5 illustrates functional enrichment modules and shows various tissue-specific functional modules dysregulated in AD and PD. We observed that 65 genes (47 upregulated, 18 downregulated) were dysregulated. These genes were involved in vascular smooth muscle cell regulation and are essential for preserving blood pressure in the brain (Ni et al., 2018). We found that inter-module interactions were predominant between endothelial cells (M2) and smooth muscle cell regulation (M6) (Figure 5). In this section, we discuss a few hubs, which are identified based on the network properties (degree > 5, detweenness centrality ≥ 0.5), and are highlighted in Figure 5.

The PSMD1 and UBE2N genes are involved in proteolysis and DNA damage response. They are downregulated in AD and PD. The QKI gene belongs to the RNA binding protein that regulates myelination. The QKI gene interacts with UBE2B and MYH10, and these genes are essential for the regulation of ubiquitination and cytoskeleton-mediated motility. These genes are downregulated and therefore show impairment of proteasome-mediated ubiquitination and cell transport processes under disease conditions. This may affect mitochondrial and vesicular mobility and hinder the quality control of mitochondria, thereby depleting ATP production. The PPM1B gene interacts with G3BP2 and participates in NF-kB regulation, which plays a key role in inflammation and apoptosis. The SIRT1 gene interacts with KAT2B, NAA16 is involved in the acetylation process, and all of these genes are upregulated under disease conditions.

In this study, the variant FLT1 (rs144398423-3′ UTR; VEGFR1) was identified in AD and PD. The FLT1 gene interacts with USF2, TAL1, ZBTB16, and GATA2, and these genes are involved in the development of the blood vessels and vasculature. The ATP-sensitive potassium channel (KCNJ2) interacts with FLT1 in the endothelial vascular pathway. Following an action potential, the potassium concentration of the extracellular region is higher, dilating the blood vessel for subsequent neuronal activity. In disease conditions, it has been observed that the dysregulation of potassium-related genes affects vasodilation through vascular smooth muscle and endothelial cell pathways. Both pathways are essential to maintain vascular integrity and the blood-brain barrier (Zenaro et al., 2017). We found that identified hub genes interact with other genes in specific biological functions from this enrichment analysis. From the above analysis, the BBB is essential for functional homeostasis of the human brain, and hence we propose that restoring the BBB may save the neuron against degeneration in both the diseases.

### Identification of Common Potential Drug Targets in AD and PD

**FIGURE 6:**
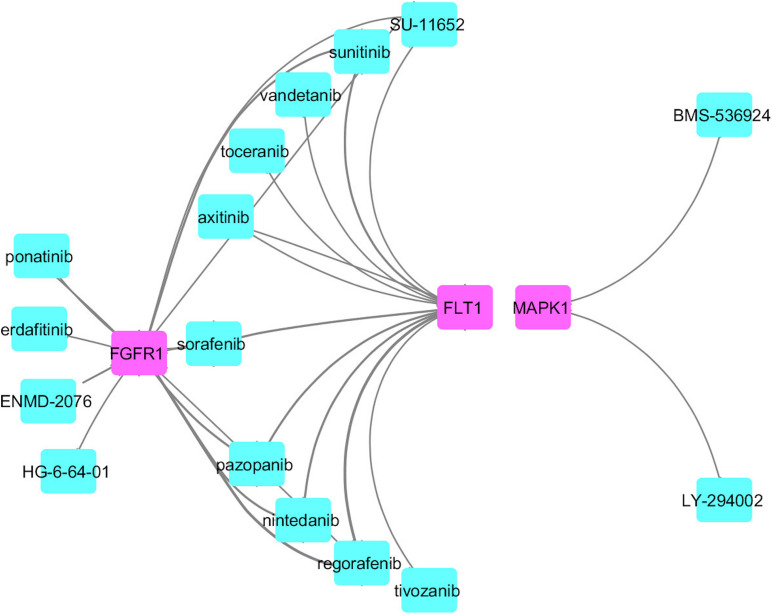
Drug connectivity map along with the proposed possible drug targets.

We identified common druggable targets to treat AD and PD disease pathogenesis and mapped the known drugs with the tissue-specific functional module network. We obtained a set of drugs based on gene expression profiles and a connectivity map, which targeted various kinase families represented in Figure 6.

**FIGURE 7:**
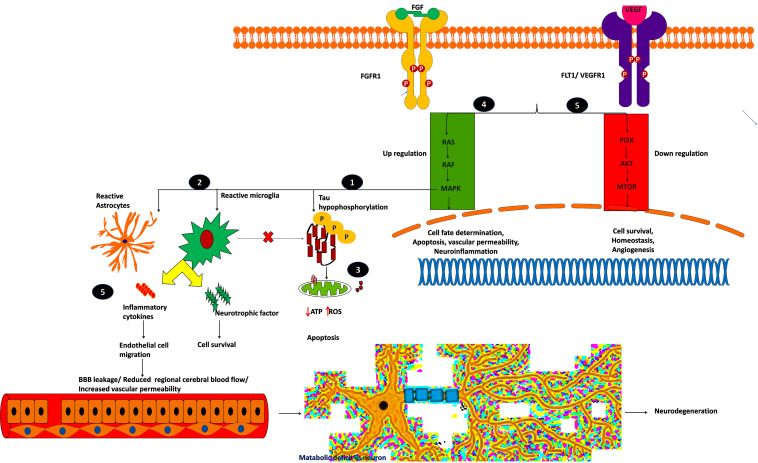
Plausible mechanism of proposed possible drug targets to reduce BBB permeability and neuroinflammation.

From this analysis, we found that potential druggable targets MAPK1, FGFR1, and FLT1 are upregulated in both AD and PD pathogenesis. The pattern of gene expression in the disease sample differs from that of other control tissues. In BA9 disease tissue, the TPM count values for these genes are high compared to control tissues (Supplementary Figure S8). Selective inhibition of these targets may provide common therapeutic interventions. However, the mapped drugs showed off-target effects and are listed in Supplementary Table S9. To understand these selected target mechanisms, we carefully mined the literature, and the details are illustrated in Figure 7.

Mitogen-activated protein kinase 1 (MAPK1), widely known as p38, participates in cell proliferation, inflammation, and stress-mediated cell death. The mechanism of proposed possible targets is explained below:

(i) MAPK1 hyper-phosphorylates Tau and converts them into insoluble aggregates, which affects synaptic transmission.
(ii) Insoluble protein aggregation activates p38 signaling which acts as a double-edged sword by releasing proinflammatory cytokines and neurotrophic factors, which may save the neuron from the insult, or it may destroy the endothelial cells by releasing cytotoxic cytokines.
(iii) Insoluble protein aggregation interrupts mitochondrial function and increases ROS production which ultimately leads to oxidative stress, which in turn activates p38 signaling and induces apoptosis (Gerschütz et al., 2014; von Bernhardi et al., 2015).

The inhibition of p38 reduces oxidative stress and provides neuroprotection by regulating autophagy (Chen et al., 2018). On the other hand, designing a specific kinase inhibitor is challenging due to its cross signaling with other kinase proteins. Most of the claimed inhibitors activate or block other kinase families (Supplementary Table S9). Further studies are required to understand and selectively target MAPK1 signaling to overcome the inflammation threat.

Fibroblast growth factor receptor-1 (FGFR1) is another potential drug target for neurodegeneration and plays an essential role in glia/endothelial cell regulation, angiogenesis, and wound healing. We found that FGFR1/FGF2 is upregulated in AD and PD.

(iv) FGFR1 provides a “help-me/eat-me” signal to microglia and astrocytes through activating p38-MAPK signaling. This signaling helps clear debris, provides neuroprotection, and provokes inflammatory cytokines, affecting the damaged neuron and endothelial cells. Further, FGFR1 expression is higher near the senile plaques of deceased patients (Ye et al., 2019; Chen et al., 2020). This shows that FGFR1 upregulation may be neuroprotective or harmful to the neuronal population. A recent study showed that after traumatic injury, FGF binds to FGFR1 and increases adhesion and tight junctions, which in turn reduces blood-brain barrier permeability and guards the neuron against chronic insult (Shi et al., 2018). The role of FGFR1 in neuroinflammation and neuroprotection needs to be elucidated through activation and antagonization of the receptor.

VEGFR1, VEGFR2, and VEGFR3 mainly regulate the VEGFR pathway and are mainly activated by permeability factor VEGF-A (Mahoney et al., 2019). This pathway is essential to regulate endothelial cell migration, blood-brain barrier permeability, cell survival, and angiogenesis. Vascular endothelial growth factor receptor-1 (VEGFR1), also denoted as FLT1, is an attractive neurovascular target for both AD and PD (Weddell et al., 2018). During hypoxic conditions, such as cerebral hypoperfusion (decreased blood pressure), VEGF-A predominantly binds to FLT1, thereby regulating angiogenesis and inflammation.

(v) The upregulated FLT1 modulates microglia migration through MAPK signaling and promotes the inflammatory response. During cell stress, the reactive astrocytes release proinflammatory cytokines and VEGF-A, which affects BBB integrity, or it may provide neurotrophic factors to overcome the insult (Patel et al., 2010; Cho et al., 2017; Zenaro et al., 2017). Yang et al. (2004) showed that increased VEGF-A expression and FLT1 form leaky blood vessels near amyloid plaques. On the other hand, plaques affect VEGF-A binding to its receptor VEGFR2 which impacts neuronal survival and vascular integrity, showing that a higher concentration of VEGF-A leads to leaky blood vessels and BBB. Further research may be needed to identify the VEGF concentration (by selectively blocking VEGFR1 signaling), reduce inflammation, and improve vasculature and BBB integrity. Selectively targeting these kinase receptors is challenging due to cross signaling with other pathways and domain similarity. It is necessary to perform further explorative experiments to evaluate these targets for therapeutic intervention to reduce BBB permeability and neuroinflammation.

## Discussion

In this study, we explored potential druggable targets for AD and PD using BA9 RNA-seq data. We identified variants and compared them with GWAS studies. We found that 167 variants are common between AD and PD, which are not present in control samples. We evaluated the effect of these GWAS variants using various *in silico* tools. A total of 128 variants are located in non-coding regions, affecting the regulatory elements involved in gene expression. The SZRD1 [rs138678090(C/T)] and YWHAB [rs188983062(G/C)] variants affect hsa-miR-301a-3p and hsa-miR-212-3p miRNA binding and these miRNAs are found to be downregulated in both diseases. The identified 3′ UTR variants disrupt the regulatory binding motif of 47 TFs, known to participate in AD and PD pathogenesis. DNMT1 and HDAC1 TFs regulate DNA methylation and histone acetylation, thereby affecting downstream signaling genes.

We built BA9 specific co-expression and functional interaction networks, and we identified the functional association between variants, DEGs, and TFs. For example, the CREBBP TF regulatory motif is disrupted by the STARD10 (rs148235301) gene variant. CREBBP activates the genes FLT1 and FGFR1, and is co-expressed in BA9; these genes are upregulated in disease conditions that are involved in vascular smooth muscle and endothelial pathways. These pathways are essential to maintain the vascular integrity and restoration of BBB and provide neurotrophic factors and metabolites to save the surviving neuronal population against various stress stimuli. Therefore, loss of BBB integrity results in disruption of neurovascular communication and cerebral blood flow (Sweeney et al., 2018b). In AD and PD, reduced regional cerebral blood flow was observed in the motor cortex (Sweeney et al., 2018a). These events led to impairment in vascular-glial communication resulting in an energy crisis and influence the energy craving neurons. Due to metabolic stress, these neurons are unable to meet their energy requirement for securing functional homeostasis. Gene signature analysis and an in-depth literature search revealed that MAPK1, FGFR1, and FLT1 are promising therapeutic targets for AD and PD.

Abnormal neuroinflammation is the biggest threat to the BBB and endothelial cells. Investigating MAPK1 and FGFR1 targets may reduce neuroinflammation and extend neuronal survival. Modulating the FLT1 pathway may provide therapeutic VEGF concentration, which may activate the VEGFR2 pathway, reducing BBB permeability and leaky blood vessels. The proposed possible targets to treat neurodegeneration belong to the kinase family. Designing selective inhibitors for the kinase family is strenuous due to its domain functionality and cross-talk between various other kinases. A recent study explains the role of the kinome and its inhibitor design can open new ventures for neuroscientists to treat CNS degeneration (Krahn et al., 2020). Exploring these targets may reduce pathological neuroinflammation and increase vasculature integrity, saving the neuron from chronic insult.

## Limitations

Major limitations of the present study are the small sample size (28 PD and nine AD samples) and the fact that we used *in silico* tools to predict the effect of variants. Although the results are reported using a small dataset all the variants identified in the work showed good agreements with other eQTL and GWAS studies of AD and PD using large datasets. The results obtained in this work could be strengthened upon the availability of a wide volume of data and experimental validation of the identified variants.

Another limitation of using pathological tissue samples is that it is not possible to distinguish targets that will address primary causative events from downstream pathology. For example, if cell type A (BA9) dies in both diseases, this would be reflected in the differential expression and appear to target this analysis. However, many possible scenarios could complicate this logic: (i) cell type A (BA9) could die via different mechanisms in these two diseases (AD/PD), making the target different (and unknown) in each condition and (ii) cell type A (BA9) might die only because cell type B has some primary malfunction that is not detected by the differential expression analysis and in this case, we would need to target cell type B.

## Data Availability Statement

The original contributions presented in the study are included in the article/Supplementary Material, further inquiries can be directed to the corresponding author/s.

## Author Contributions

MMG and SAPD conceived the project. SAPD carried out the computations. SAPD, SJ, YT, and MMG contributed toward discussions and manuscript preparation. All authors read and finalized the manuscript.

## Conflict of Interest

The authors declare that the research was conducted in the absence of any commercial or financial relationships that could be construed as a potential conflict of interest.

## References

[B1] Abbott N. J. (2002). Astrocyte-endothelial interactions and blood-brain barrier permeability. *J. Anat.* 200 629–638. 10.1046/J.1469-7580.2002.00064.X 12162730 PMC1570746

[B2] Alanis-Lobato G. Andrade-Navarro M. A. Schaefer M. H. (2017). HIPPIE v2.0: enhancing meaningfulness and reliability of protein-protein interaction networks. *Nucleic Acids Res.* 45 D408–D414. 10.1093/nar/gkw985 27794551 PMC5210659

[B3] Albert F. W. Kruglyak L. (2015). The role of regulatory variation in complex traits and disease. *Nat. Rev. Genet.* 16 197–212. 10.1038/nrg3891 25707927

[B4] Angelova M. T. Dimitrova D. G. Dinges N. Lence T. Worpenberg L. Carré C. (2018). The emerging field of epitranscriptomics in neurodevelopmental and neuronal disorders. *Front. Bioeng. Biotechnol.* 6:46. 10.3389/fbioe.2018.00046 29707539 PMC5908907

[B5] Araujo P. R. Yoon K. Ko D. Smith A. D. Qiao M. Suresh U. (2012). Before it gets started: regulating translation at the 5’ UTR. *Comp. Funct. Genomics* 2012 1–8. 10.1155/2012/475731 22693426 PMC3368165

[B6] Bélanger M. Allaman I. Magistretti P. J. (2011). Brain energy metabolism: focus on astrocyte-neuron metabolic cooperation. *Cell Metab.* 14 724–738. 10.1016/J.CMET.2011.08.016 22152301

[B7] Berchtold N. C. Prieto G. A. Phelan M. Gillen D. L. Baldi P. Bennett D. A. (2019). Hippocampal gene expression patterns linked to late-life physical activity oppose age and AD-related transcriptional decline. *Neurobiol. Aging* 78 142–154. 10.1016/j.neurobiolaging.2019.02.012 30927700 PMC6901108

[B8] Blalock E. M. Geddes J. W. Chen K. C. Porter N. M. Markesbery W. R. Landfield P. W. (2004). Incipient Alzheimer’s disease: microarray correlation analyses reveal major transcriptional and tumor suppressor responses. *Proc. Natl. Acad. Sci. U.S.A.* 101 2173–2178. 10.1073/pnas.0308512100 14769913 PMC357071

[B9] Borrageiro G. Haylett W. Seedat S. Kuivaniemi H. Bardien S. (2018). A review of genome-wide transcriptomics studies in Parkinson’s disease. *Eur. J. Neurosci.* 47 1–16. 10.1111/ejn.13760 29068110

[B10] Carithers L. J. Moore H. M. (2015). The Genotype-Tissue Expression (GTEx) Project. *Biopreserv. Biobank.* 13 307–308. 10.1089/bio.2015.29031.hmm 26484569 PMC4692118

[B11] Chen J. Ren Y. Gui C. Zhao M. Wu X. Mao K. (2018). Phosphorylation of Parkin at serine 131 by p38 MAPK promotes mitochondrial dysfunction and neuronal death in mutant A53T α-synuclein model of Parkinson’s disease. *Cell Death Dis.* 9:700. 10.1038/s41419-018-0722-7 29899409 PMC5999948

[B12] Chen P. Tang H. Zhang Q. Xu L. Zhou W. Hu X. (2020). Basic fibroblast growth factor (bfgf) protects the blood-brain barrier by binding of FGFR1 and activating the ERK signaling pathway after intra-abdominal hypertension and traumatic brain injury. *Med. Sci. Monit.* 26:e922009. 10.12659/MSM.922009 32036381 PMC7029819

[B13] Chen Y. Wang X. (2020). MiRDB: an online database for prediction of functional microRNA targets. *Nucleic Acids Res.* 48 D127–D131. 10.1093/nar/gkz757 31504780 PMC6943051

[B14] Cho S. J. Park M. H. Han C. Yoon K. Koh Y. H. (2017). VEGFR2 alteration in Alzheimer’s disease. *Sci. Rep.* 7:17713. 10.1038/s41598-017-18042-1 29255164 PMC5735090

[B15] Ciryam P. Kundra R. Freer R. Morimoto R. I. Dobson C. M. Vendruscolo M. (2016). A transcriptional signature of Alzheimer’s disease is associated with a metastable subproteome at risk for aggregation. *Proc. Natl. Acad. Sci. U.S.A.* 113 4753–4758. 10.1073/pnas.1516604113 27071083 PMC4855616

[B16] CoppedÃ F. (2014). The potential of epigenetic therapies in neurodegenerative diseases. *Front. Genet.* 5:220. 10.3389/fgene.2014.00220 25071843 PMC4094885

[B17] Craft S. (2007). Insulin resistance and Alzheimer’s disease pathogenesis: potential mechanisms and implications for treatment. *Curr. Alzheimer Res.* 4 147–152. 10.2174/156720507780362137 17430239

[B18] Damier P. Hirsch E. C. Agid Y. Graybiel A. M. (1999). The substantia nigra of the human brain. *Brain* 122 1437–1448. 10.1093/brain/122.8.1437 10430830

[B19] Dayem Ullah A. Z. Oscanoa J. Wang J. Nagano A. Lemoine N. R. Chelala C. (2018). SNPnexus: assessing the functional relevance of genetic variation to facilitate the promise of precision medicine. *Nucleic Acids Res.* 46 W109–W113. 10.1093/nar/gky399 29757393 PMC6030955

[B20] DeKosky S. T. Scheff S. W. (1990). Synapse loss in frontal cortex biopsies in Alzheimer’s disease: correlation with cognitive severity. *Ann. Neurol.* 27 457–464. 10.1002/ana.410270502 2360787

[B21] Deming Y. Li Z. Kapoor M. Harari O. Del-Aguila J. L. Black K. (2017). Genome-wide association study identifies four novel loci associated with Alzheimer’s endophenotypes and disease modifiers. *Acta Neuropathol.* 133 839–856. 10.1007/s00401-017-1685-y 28247064 PMC5613285

[B22] Dharshini S. A. P. Taguchi Y.-H. Gromiha M. M. (2019). Investigating the energy crisis in Alzheimer disease using transcriptome study. *Sci. Rep.* 9:18509. 10.1038/s41598-019-54782-y 31811163 PMC6898285

[B23] Dobin A. Davis C. A. Schlesinger F. Drenkow J. Zaleski C. Jha S. (2013). STAR: ultrafast universal RNA-seq aligner. *Bioinformatics* 29 15–21. 10.1093/bioinformatics/bts635 23104886 PMC3530905

[B24] Donega V. Burm S. M. van Strien M. E. van Bodegraven E. J. Paliukhovich I. Geut H. (2019). Transcriptome and proteome profiling of neural stem cells from the human subventricular zone in Parkinson’s disease. *Acta Neuropathol. Commun.* 7:84. 10.1186/s40478-019-0736-0 31159890 PMC6545684

[B25] Dumitriu A. Golji J. Labadorf A. T. Gao B. Beach T. G. Myers R. H. (2016). Integrative analyses of proteomics and RNA transcriptomics implicate mitochondrial processes, protein folding pathways and GWAS loci in Parkinson disease. *BMC Med. Genomics* 9:5. 10.1186/s12920-016-0164-y 26793951 PMC4722694

[B26] Freeman L. R. Keller J. N. (2012). Oxidative stress and cerebral endothelial cells: regulation of the blood-brain-barrier and antioxidant based interventions. *Biochim. Biophys. Acta* 1822 822–829. 10.1016/j.bbadis.2011.12.009 22206999 PMC3412391

[B27] Gan L. Cookson M. R. Petrucelli L. La Spada A. R. (2018). Converging pathways in neurodegeneration, from genetics to mechanisms. *Nat. Neurosci.* 21 1300–1309. 10.1038/s41593-018-0237-7 30258237 PMC6278826

[B28] Gerschütz A. Heinsen H. Grünblatt E. Wagner A. K. Bartl J. Meissner C. (2014). Neuron-specific alterations in signal transduction pathways associated with Alzheimer’s disease. *J. Alzheimers. Dis.* 40 135–142. 10.3233/JAD-131280 24334724

[B29] Greene C. S. Krishnan A. Wong A. K. Ricciotti E. Zelaya R. A. Himmelstein D. S. (2015). Understanding multicellular function and disease with human tissue-specific networks. *Nat. Genet.* 47 569–576. 10.1038/ng.3259 25915600 PMC4828725

[B30] Guttula S. V. Allam A. Gumpeny R. S. (2012). Analyzing microarray data of Alzheimer’s using cluster analysis to identify the biomarker genes. *Int. J. Alzheimers. Dis.* 2012:649456. 10.1155/2012/649456 22482075 PMC3296213

[B31] Huang D. Yi X. Zhang S. Zheng Z. Wang P. Xuan C. (2018). GWAS4D: multidimensional analysis of context-specific regulatory variant for human complex diseases and traits. *Nucleic Acids Res.* 46 W114–W120. 10.1093/nar/gky407 29771388 PMC6030885

[B32] Jun G. R. Chung J. Mez J. Barber R. Beecham G. W. Bennett D. A. (2017). Transethnic genome-wide scan identifies novel Alzheimer’s disease loci. *Alzheimer’s Dement.* 13 727–738. 10.1016/J.JALZ.2016.12.012 28183528 PMC5496797

[B33] Karagulle A. T. Lehericy K. S. Luciana M. Ugurbil K. Tuite P. (2008). Altered Diffusion in the Frontal Lobe in Parkinson Disease. *AJNR Am. J. Neuroradiol.* 29 501–505. 10.3174/ajnr.A0850 18202242 PMC8118887

[B34] Krahn A. I. Wells C. Drewry D. H. Beitel L. K. Durcan T. M. Axtman A. D. (2020). Defining the neural kinome: strategies and opportunities for small molecule drug discovery to target neurodegenerative diseases. *ACS Chem. Neurosci.* 11 1871–1886. 10.1021/acschemneuro.0c00176 32464049

[B35] Krueger F. (2015). *Trim Galore!: A wrapper tool around Cutadapt and FastQC to consistently apply quality and adapter trimming to FastQ files.* Cambridge, MA: The Babraham Institute.

[B36] Kumar S. Ambrosini G. Bucher P. (2017). SNP2TFBS - a database of regulatory SNPs affecting predicted transcription factor binding site affinity. *Nucleic Acids Res.* 45 D139–D144. 10.1093/nar/gkw1064 27899579 PMC5210548

[B37] Kumar S. Reddy P. H. (2016). Are circulating microRNAs peripheral biomarkers for Alzheimer’s disease? *Biochim. Biophys. Acta - Mol. Basis Dis.* 1862 1617–1627. 10.1016/j.bbadis.2016.06.001 27264337 PMC5343750

[B38] Lamb J. Crawford E. D. Peck D. Modell J. W. Blat I. C. Wrobel M. J. (2006). The connectivity map: using gene-expression signatures to connect small molecules, genes, and disease. *Science* 313 1929–1935. 10.1126/science.1132939 17008526

[B39] Lambert J. C. Ibrahim-Verbaas C. A. Harold D. Naj A. C. Sims R. Bellenguez C. (2013). Meta-analysis of 74,046 individuals identifies 11 new susceptibility loci for Alzheimer’s disease. *Nat. Genet.* 45 1452–1458. 10.1038/ng.2802 24162737 PMC3896259

[B40] Lanke V. Moolamalla S. T. R. Roy D. Vinod P. K. (2018). Integrative analysis of hippocampus gene expression profiles identifies network alterations in aging and Alzheimer’s disease. *Front. Aging Neurosci.* 10:153. 10.3389/fnagi.2018.00153 29875655 PMC5974201

[B41] Lasky J. L. Wu H. (2005). Notch signaling, brain development, and human disease. *Pediatr. Res.* 57 104R–109R. 10.1203/01.PDR.0000159632.70510.3D 15817497

[B42] Lau S. F. Cao H. Fu A. K. Y. Ip N. Y. (2020). Single-nucleus transcriptome analysis reveals dysregulation of angiogenic endothelial cells and neuroprotective glia in Alzheimer’s disease. *Proc. Natl. Acad. Sci. U.S.A.* 117 25800–25809. 10.1073/pnas.2008762117 32989152 PMC7568283

[B43] Lee S. Zhang C. Arif M. Liu Z. Benfeitas R. Bidkhori G. (2018). TCSBN: a database of tissue and cancer specific biological networks. *Nucleic Acids Res.* 46 D595–D600. 10.1093/nar/gkx994 29069445 PMC5753183

[B44] Lesnick T. G. Papapetropoulos S. Mash D. C. Ffrench-Mullen J. Shehadeh L. De Andrade M. (2007). A genomic pathway approach to a complex disease: axon guidance and Parkinson disease. *PLoS Genet.* 3:e0030098. 10.1371/journal.pgen.0030098 17571925 PMC1904362

[B45] Levine A. J. Miller J. A. Shapshak P. Gelman B. Singer E. J. Hinkin C. H. (2013). Systems analysis of human brain gene expression: mechanisms for HIV-associated neurocognitive impairment and common pathways with Alzheimer’s disease. *BMC Med. Genomics* 6:4. 10.1186/1755-8794-6-4 23406646 PMC3626801

[B46] Liang W. S. Reiman E. M. Valla J. Dunckley T. Beach T. G. Grover A. (2008). Alzheimer’s disease is associated with reduced expression of energy metabolism genes in posterior cingulate neurons. *Proc. Natl. Acad. Sci. U.S.A.* 105 4441–4446. 10.1073/pnas.0709259105 18332434 PMC2393743

[B47] Love M. I. Huber W. Anders S. (2014). Moderated estimation of fold change and dispersion for RNA-seq data with DESeq2. *Genome Biol.* 15:550. 10.1186/s13059-014-0550-8 25516281 PMC4302049

[B48] Ma M. Ru Y. Chuang L. S. Hsu N. Y. Shi L. S. Hakenberg J. (2015). Disease-associated variants in different categories of disease located in distinct regulatory elements. *BMC Genomics* 16:S3. 10.1186/1471-2164-16-S8-S3 26110593 PMC4480828

[B49] Mahoney E. R. Dumitrescu L. Moore A. M. Cambronero F. E. De Jager P. L. Koran M. E. I. (2019). Brain expression of the vascular endothelial growth factor gene family in cognitive aging and alzheimer’s disease. *Mol. Psychiatry* 26 888–896. 10.1038/s41380-019-0458-5 31332262 PMC6980445

[B50] Mao F. Xiao L. Li X. Liang J. Teng H. Cai W. (2016). RBP-var: a database of functional variants involved in regulation mediated by RNA-binding proteins. *Nucleic Acids Res.* 44 D154–D163. 10.1093/nar/gkv1308 26635394 PMC4702914

[B51] McGuire M. H. Herbrich S. M. Dasari S. K. Wu S. Y. Wang Y. Rupaimoole R. (2019). Pan-cancer genomic analysis links 3’UTR DNA methylation with increased gene expression in T cells. *EBioMedicine* 43 127–137. 10.1016/j.ebiom.2019.04.045 31056473 PMC6558231

[B52] McKenna A. Hanna M. Banks E. Sivachenko A. Cibulskis K. Kernytsky A. (2010). The genome analysis Toolkit: a MapReduce framework for analyzing next-generation DNA sequencing data. *Genome Res.* 20 1297–1303. 10.1101/gr.107524.110 20644199 PMC2928508

[B53] Muddapu V. R. Dharshini S. A. P. Chakravarthy V. S. Gromiha M. M. (2020). Neurodegenerative diseases – is metabolic deficiency the root cause? *Front. Neurosci.* 14:213. 10.3389/fnins.2020.00213 32296300 PMC7137637

[B54] Narayanan M. Huynh J. L. Wang K. Yang X. Yoo S. McElwee J. (2014). Common dysregulation network in the human prefrontal cortex underlies two neurodegenerative diseases. *Mol. Syst. Biol.* 10:743. 10.15252/msb.20145304 25080494 PMC4299500

[B55] Ng B. White C. C. Klein H.-U. Sieberts S. K. McCabe C. Patrick E. (2017). An xQTL map integrates the genetic architecture of the human brain’s transcriptome and epigenome. *Nat. Neurosci.* 20 1418–1426. 10.1038/nn.4632 28869584 PMC5785926

[B56] Ni R. Rudin M. Klohs J. (2018). Cortical hypoperfusion and reduced cerebral metabolic rate of oxygen in the arcAβ mouse model of Alzheimer’s disease. *Photoacoustics* 10 38–47. 10.1016/j.pacs.2018.04.001 29682448 PMC5909030

[B57] Pallarès-Albanell J. Teresa Zomeño-Abellán M. Escaramís G. Pantano L. Soriano A. Segura M. F. (2019). A high-throughput screening identifies MicroRNA inhibitors that influence neuronal maintenance and/or response to oxidative stress. *Mol. Ther. Nucleic Acid* 17 374–387. 10.1016/j.omtn.2019.06.007 31302497 PMC6626867

[B58] Pankratz N. Beecham G. W. DeStefano A. L. Dawson T. M. Doheny K. F. Factor S. A. (2012). Meta-analysis of Parkinson’s disease: identification of a novel locus, RIT2. *Ann. Neurol.* 71 370–384. 10.1002/ana.22687 22451204 PMC3354734

[B59] Patel H. Hodges A. K. Curtis C. Lee S. H. Troakes C. Dobson R. J. B. (2019). Transcriptomic analysis of probable asymptomatic and symptomatic alzheimer brains. *Brain Behav. Immun.* 80 644–656. 10.1016/j.bbi.2019.05.009 31063847

[B60] Patel N. S. Mathura V. S. Bachmeier C. Beaulieu-Abdelahad D. Laporte V. Weeks O. (2010). Alzheimer’s β-amyloid peptide blocks vascular endothelial growth factor mediated signaling via direct interaction with VEGFR-2. *J. Neurochem.* 112 66–76. 10.1111/j.1471-4159.2009.06426.x 19818105

[B61] Patro R. Duggal G. Love M. I. Irizarry R. A. Kingsford C. (2017). Salmon provides fast and bias-aware quantification of transcript expression. *Nat. Methods* 14 417–419. 10.1038/nmeth.4197 28263959 PMC5600148

[B62] Piehl F. Olsson T. (2009). Inflammation and susceptibility to neurodegeneration: the use of unbiased genetics to decipher critical regulatory pathways. *Neuroscience* 158 1143–1150. 10.1016/j.neuroscience.2008.08.031 18805461

[B63] Piras I. S. Krate J. Delvaux E. Nolz J. Mastroeni D. F. Persico A. M. (2019). Transcriptome changes in the Alzheimer’s disease middle temporal gyrus: importance of RNA metabolism and mitochondria-associated membrane genes. *J. Alzheimer’s Dis.* 70 691–713. 10.3233/JAD-181113 31256118

[B64] Raj T. Li Y. I. Wong G. Humphrey J. Wang M. Ramdhani S. (2018). Integrative transcriptome analyses of the aging brain implicate altered splicing in Alzheimer’s disease susceptibility. *Nat. Genet.* 50 1584–1592. 10.1038/s41588-018-0238-1 30297968 PMC6354244

[B65] Scheckel C. Drapeau E. Frias M. A. Park C. Y. Fak J. Zucker-Scharff I. (2016). Regulatory consequences of neuronal ELAV-like protein binding to coding and non-coding RNAs in human brain. *eLife* 5:e10421. 10.7554/eLife.10421 26894958 PMC4798961

[B66] Shi Y.-J. Shi M. Xiao L.-J. Li L. Zou L.-H. Li C.-Y. (2018). Inhibitive effects of FGF2/FGFR1 pathway on astrocyte-mediated inflammation in vivo and in vitro after infrasound exposure. *Front. Neurosci.* 12:582. 10.3389/fnins.2018.00582 30210273 PMC6119807

[B67] Siepel A. Bejerano G. Pedersen J. S. Hinrichs A. S. Hou M. Rosenbloom K. (2005). Evolutionarily conserved elements in vertebrate, insect, worm, and yeast genomes. *Genome Res.* 15 1034–1050. 10.1101/gr.3715005 16024819 PMC1182216

[B68] Simchovitz A. Hanan M. Yayon N. Lee S. Bennett E. R. Greenberg D. S. (2020). A lncRNA survey finds increases in neuroprotective LINC-PINT in Parkinson’s disease substantia nigra. *Aging Cell* 19:13115. 10.1111/acel.13115 32080970 PMC7059180

[B69] Srinivasan K. Friedman B. A. Etxeberria A. Huntley M. A. van der Brug M. P. Foreman O. (2020). Alzheimer’s patient microglia exhibit enhanced aging and unique transcriptional activation. *Cell Rep.* 31:107843. 10.1016/j.celrep.2020.107843 32610143 PMC7422733

[B70] Stathias V. Turner J. Koleti A. Vidovic D. Cooper D. Fazel-Najafabadi M. (2020). LINCS Data Portal 2.0: next generation access point for perturbation-response signatures. *Nucleic Acids Res.* 48 D431–D439. 10.1093/nar/gkz1023 31701147 PMC7145650

[B71] Steri M. Idda M. L. Whalen M. B. Orrù V. (2018). Genetic variants in mRNA untranslated regions. *Wiley Interdiscip. Rev. RNA* 9:e1474. 10.1002/wrna.1474 29582564 PMC6002891

[B72] Subramanian A. Narayan R. Corsello S. M. Peck D. D. Natoli T. E. Lu X. (2017). A next generation connectivity map: L1000 platform and the first 1,000,000 profiles. *Cell* 171 1437.e17–1452.e17. 10.1016/j.cell.2017.10.049 29195078 PMC5990023

[B73] Sweeney M. D. Kisler K. Montagne A. Toga A. W. Zlokovic B. V. (2018a). The role of brain vasculature in neurodegenerative disorders. *Nat. Neurosci.* 21 1318–1331. 10.1038/s41593-018-0234-x 30250261 PMC6198802

[B74] Sweeney M. D. Sagare A. P. Zlokovic B. V. (2018b). Blood–brain barrier breakdown in Alzheimer disease and other neurodegenerative disorders. *Nat. Rev. Neurol.* 14 133–150. 10.1038/nrneurol.2017.188 29377008 PMC5829048

[B75] Tiwari V. Patel A. B. (2014). Pyruvate carboxylase and pentose phosphate fluxes are reduced in AβPP-PS1 mouse model of Alzheimer’s disease: a 13C NMR study. *J. Alzheimer’s Dis.* 41 387–399. 10.3233/JAD-122449 24625793

[B76] Valor L. Viosca J. Lopez-Atalaya J. Barco A. (2013). Lysine acetyltransferases CBP and p300 as therapeutic targets in cognitive and neurodegenerative disorders. *Curr. Pharm. Des.* 19 5051–5064. 10.2174/13816128113199990382 23448461 PMC3722569

[B77] von Bernhardi R. Cornejo F. Parada G. E. Eugenín J. (2015). Role of TGFβ signaling in the pathogenesis of Alzheimer’s disease. *Front. Cell. Neurosci.* 9:426. 10.3389/fncel.2015.00426 26578886 PMC4623426

[B78] Wang H. Lou D. Wang Z. (2019). Crosstalk of genetic variants, allele-specific DNA methylation, and environmental factors for complex disease risk. *Front. Genet.* 10:695. 10.3389/fgene.2018.00695 30687383 PMC6334214

[B79] Wang J. Raskin L. Samuels D. C. Shyr Y. Guo Y. (2015). Genome measures used for quality control are dependent on gene function and ancestry. *Bioinformatics* 31 318–323. 10.1093/bioinformatics/btu668 25297068 PMC4308666

[B80] Wang M. Roussos P. McKenzie A. Zhou X. Kajiwara Y. Brennand K. J. (2016). Integrative network analysis of nineteen brain regions identifies molecular signatures and networks underlying selective regional vulnerability to Alzheimer’s disease. *Genome Med.* 8:104. 10.1186/s13073-016-0355-3 27799057 PMC5088659

[B81] Wang X. Michaelis E. K. (2010). Selective neuronal vulnerability to oxidative stress in the brain. *Front. Aging Neurosci.* 2:12. 10.3389/fnagi.2010.00012 20552050 PMC2874397

[B82] Wang X. Pal R. Chen X. W. Kumar K. N. Kim O. J. Michaelis E. K. (2007). Genome-wide transcriptome profiling of region-specific vulnerability to oxidative stress in the hippocampus. *Genomics* 90 201–212. 10.1016/j.ygeno.2007.03.007 17553663 PMC2065755

[B83] Ward L. D. Kellis M. (2012). HaploReg: a resource for exploring chromatin states, conservation, and regulatory motif alterations within sets of genetically linked variants. *Nucleic Acids Res.* 40 D930–D934. 10.1093/nar/gkr917 22064851 PMC3245002

[B84] Weddell J. C. Chen S. Imoukhuede P. I. (2018). VEGFR1 promotes cell migration and proliferation through PLCγ and PI3K pathways. *NPJ Syst. Biol. Appl.* 4 1–11. 10.1038/s41540-017-0037-9 29263797 PMC5736688

[B85] Wilde G. J. Pringle A. K. Wright P. Iannotti F. (1997). Differential vulnerability of the CA1 and CA3 subfields of the hippocampus to superoxide and hydroxyl radicals in vitro. *J. Neurochem.* 69 883–886. 10.1046/j.1471-4159.1997.69020883.x 9231752

[B86] Wu G. Dawson E. Duong A. Haw R. Stein L. (2014). ReactomeFIViz: a Cytoscape app for pathway and network-based data analysis. *F1000Research* 3:146. 10.12688/f1000research.4431.2 25309732 PMC4184317

[B87] Yang S. P. Bae D. G. Kang H. J. Gwag B. J. Gho Y. S. Chae C. B. (2004). Co-accumulation of vascular endothelial growth factor with β-amyloid in the brain of patients with Alzheimer’s disease. *Neurobiol. Aging* 25 283–290. 10.1016/S0197-4580(03)00111-8 15123332

[B88] Ye L. Wang X. Cai C. Zeng S. Bai J. Guo K. (2019). FGF21 promotes functional recovery after hypoxic-ischemic brain injury in neonatal rats by activating the PI3K/Akt signaling pathway via FGFR1/β-klotho. *Exp. Neurol.* 317 34–50. 10.1016/j.expneurol.2019.02.013 30802446

[B89] Zenaro E. Piacentino G. Constantin G. (2017). The blood-brain barrier in Alzheimer’s disease. *Neurobiol. Dis.* 107 41–56. 10.1016/J.NBD.2016.07.007 27425887 PMC5600438

[B90] Zheng B. Liao Z. Locascio J. J. Lesniak K. A. Roderick S. S. Watt M. L. (2010). PGC-1α, a potential therapeutic target for early intervention in Parkinson’s disease. *Sci. Transl. Med.* 2:52ra73. 10.1126/scitranslmed.3001059 20926834 PMC3129986

