# Supplementary material for: Exploring Common Therapeutic Targets for Neurodegenerative Disorders Using Transcriptome Study
Source: Front Genet. 2021 Mar 19;12:639160. doi: 10.3389/fgene.2021.639160 (PMC8017312; doi:10.3389/fgene.2021.639160)
Supplement: Supplementary file 1 [file Data_Sheet_1.zip › Supplementary Tables &_Figures.pdf]

Supplementary Tables

Exploring common therapeutic targets for neurodegeneration using transcriptome study

S. Akila Parvathy Dharshini[1], Sherlyn Jemimah[1], Y-h. Taguchi[2] and M. Michael Gromiha[1,*]

Table S1: Dataset for RNA-seq analysis

**Sequence Retrieval project ID**	**Number of Control**	**Number of Patients**	**Tissue location**
SRP034831 (AD)	8	9	BA9
SRP058181 (PD)	44	28	BA9

Table S2: Genome-Wide-Association-Study of Alzheimer and Parkinson disease.

Consortium/niagads ID	Source of Sample	Sample size
NG00088	Blood	3784 control, 1,825 AD
NG00075	Blood	10,483 control, 8,362 AD
NG00073	Blood	3444 control, 2,386 AD
NG00058	Blood	25,849 control, 14,406 AD
NG00056	Blood	20,474 control, 5,813 AD
NG00055	CSF	3146 Control, 3,154 AD
NG00053	Blood	11,312 control, 8,572 AD
NG00052	Blood	520 control, 1116 AD
NG00050	CSF/plasma	934 control, 1094 AD
dbGap	Blood	4238 PD, 4239 control

AD: Alzheimer disease, PD: Parkinson disease

Table S3: Nucleotide frequency matrix for human genome.

	A	G	C	T
Intronic	0.279349	0.201834	0.211026	0.30779
exonic	0.261633	0.257771	0.261972	0.218623
upstream	0.241386	0.256568	0.257589	0.244457
UTR3	0.272023	0.216989	0.219109	0.291879
UTR5	0.234958	0.263271	0.271271	0.230501

Table S4: The frequency of nucleotide in various genomic locations.

	intronic	exonic	Upstream	UTR3	UTR5
Control variants	**A->G,T->C**	T->C,A->G	T->C,G->A	T>C, A->G	C->T,T->C,T->G
AD/PD (BA9)	**C->T, G->A**	G->A,C->T	G->A	G->A,T->C	T->C
GWAS (AD/PD)	**G->A,C->T**	A->G,T->C	C->T,A->G	T->C,A->G	A->G

control variants	**A->G**	A->T	A->C	G->A	G->C	G->T	C->T	C->G	C->A	**T->C**	T->G	T->A
intronic	**0.83**	0.07	0.13	0.59	0.22	0.14	0.66	0.24	0.16	**0.79**	0.12	0.06
exonic	0.82	0.06	0.08	0.80	0.17	0.09	0.28	0.28	0.17	1.08	0.20	0.03
Upstream	0.66	0.00	0.17	0.78	0.16	0.16	0.70	0.16	0.23	0.90	0.08	0.00
UTR3	0.60	0.09	0.17	0.50	0.21	0.21	0.63	0.39	0.21	0.66	0.13	0.05
UTR5	0.50	0.25	0.25	0.22	0.00	0.45	0.69	0.46	0.23	0.51	0.51	0.00
AD/PD (BA9)	A->G	A->T	A->C	**G->A**	G->C	G->T	**C->T**	C->G	C->A	T->C	T->G	T->A
intronic	0.02	0.00	0.00	**0.03**	0.01	0.00	**0.04**	0.01	0.01	0.02	0.00	0.00
exonic	0.06	0.03	0.00	0.40	0.00	0.03	0.39	0.06	0.03	0.10	0.00	0.03
Upstream	0.00	0.00	0.00	0.16	0.00	0.00	0.00	0.00	0.00	0.00	0.00	0.00
UTR3	0.26	0.03	0.03	0.68	0.11	0.07	0.24	0.12	0.12	0.35	0.03	0.05
UTR5	0.00	0.00	0.00	0.00	0.22	0.00	0.00	0.00	0.23	0.26	0.00	0.00
GWAS(AD/PD)	A->G	A->T	A->C	**G->A**	G->C	G->T	**C->T**	C->G	C->A	T->C	T->G	T->A
intronic	1.47	0.24	0.35	**1.98**	0.53	0.46	**1.76**	0.57	0.43	1.40	0.30	0.23
exonic	0.67	0.06	0.06	0.20	0.06	0.06	0.36	0.17	0.06	0.61	0.10	0.13
Upstream	6.38	0.91	1.66	6.24	1.64	1.33	6.60	1.63	1.32	5.81	1.06	0.82
UTR3	0.57	0.06	0.06	0.54	0.25	0.18	0.51	0.12	0.27	0.66	0.08	0.05
UTR5	0.50	0.00	0.25	0.45	0.22	0.00	0.23	0.46	0.23	0.26	0.00	0.00

Table S5: Identified variant (AD, PD) effect on Regulatory binding motifs (TF,miRNA,RNA binding protein									
Chr	Variant Position	Ref/Alt	Genomic Location	Variant Gene	dbSNP	Affected Regulatory element (TF/miRNA)	AD (log_2FC)	PD (log2 FC)	RNA binding motif effect
11	67397602	G/A	UTR3	RAD9A	rs191668067	IRF5	-2.6	-3.2	RBP motif+ribosnitch+RBP binding
1	107571722	A/G	UTR3	VAV3	rs17236120	BCL11A	8	6.7	ribosnitch+RBP binding
1	112670362	C/T	UTR3	CAPZA1	rs72984526	STAT2	-3.4	-4.8	RBP motif+ribosnitch+RBP binding+miRNA target
1	211374024	C/A	UTR3	TRAF5	rs58510761	SMARCA1	3.9	4.2	ribosnitch+RBP binding
2	127736411	A/G	UTR3	WDR33	rs72968953	JMJD6	-5.8	-7.1	-
2	130370301	C/T	intronic	PTPN18	rs115044783	TAL1	-4	-5.3	-
2	223978684	T/C	intronic	SERPINE2	rs12475117	CUX1	2.3	1.0	-
2	227325328	C/A	UTR5	MFF	rs145568471	SETDB1	-5.9	-7.2	ribosnitch+RBP binding
2	240524222	C/T	exonic	ANKMY1	rs3796119	BCL11A	8	6.7	-
2	74461951	C/T	exonic	MOGS	rs142032474	TAL1	-4	-5.3	-
2	79965496	A/G	intronic	CTNNA2	rs116603385	NCOR1	-2.5	-3.8	-
3	113274445	C/T	exonic	BOC	rs13093741	DNMT1	5.4	3.8	-
3	140977583	G/A	UTR3	SLC25A36	rs73234808	ZBTB1	-2.7	-4.0	-
3	170891152	G/A	UTR3	EIF5A2	rs144440280	STAT3	-3.4	-2.7	-
3	185080309	G/A	UTR3	C3orf70	rs113805911	BCL11A	8	6.7	-
3	47001748	C/T	exonic	NBEAL2	rs12489851	DNMT1	5.4	3.8	-
3	47009536	A/G	UTR3	NBEAL2	rs2305640	NR2C1	2.9	1.6	-
4	959671	G/A	UTR3	DGKQ	rs75067698	DNMT1	5.4	3.8	-
5	150123275	G/C	intronic	PDGFRB	rs56388645	NFYA	-5.5	-6.8	-
5	150650496	C/T	UTR3	SYNPO	rs113450445	NANOG	2.9	1.6	-
5	151268175	G/C	UTR3	GM2A	rs112641014	NCOR1	-2.5	-3.8	-
5	168428039	A/G	exonic	WWC1	rs61730019	CUX1	2.3	1.0	-
5	169601510	A/T	exonic	SPDL1	rs144151613	E2F1	-3.5	-2.1	-

5	6668802	G/A	UTR3	SRD5A1	rs1042150	KAT2B	6.8	2.7	RBP motif+ribosnitch+RBP binding+miRNA target
6	170581705	C/A	intronic	PDCD2	rs12212565	MLLT1	-4.4	-5.7	-
6	40399118	G/A	intronic	LRFN2	rs74613782	GATA2	-3.5	-4.8	-
6	75918300	T/C	UTR3	MYO6	rs118121148	SRF	-5.3	-3.1	-
6	99432504	A/G	UTR3	USP45	rs1134718	CUX1	2.3	1.0	ribosnitch+RBP binding
7	1058205	G/A	exonic	GPR146	rs61976951	DNMT1	5.4	3.8	-
7	19696657	T/C	UTR3	TWISTNB	rs17354985	MEF2A	2.7	1.4	-
7	26199466	G/C	intronic	HNRNPA2B1	rs55657742	HOXA1	-3.8	-5.1	-
7	30083529	A/G	UTR3	PLEKHA8	rs17158618	TCF12	4	2.7	-
7	47368702	G/A	exonic	TNS3	rs76229426	IRF5	-2.6	-3.2	-
7	76273420	G/A	intronic	SRRM3	rs77373389	ZBTB7A	8.7	3.7	-
7	76276343	G/A	intronic	SRRM3	rs73373453	SOX2	10.8	5.3	-
8	101689173	G/A	UTR3	NCALD	rs113375628	DNMT1	5.4	3.8	-
8	107900534	A/G	UTR3	RSPO2	rs117326891	SRF	-5.3	-3.1	-
8	117539603	T/C	intronic	MED30	rs72672139	TAL1	-4	-5.3	-
8	142276957	C/G	intronic	TSNARE1	rs76873068	SETDB1	-5.9	-7.2	-
8	22096506	C/T	exonic	FAM160B2	rs117838157	DNMT1	5.4	3.8	ribosnitch+RBP binding
9	15468045	A/G	intronic	PSIP1		HDAC1	-4.7	-3.5	-
9	33042617	C/T	UTR3	SMU1	rs41274007	RFX5	-4.4	-5.7	-
9	89472148	A/G	intronic	SEMA4D	rs59446279	JUN	-3.2	-4.5	-
9	92613432	G/A	UTR3	CENPP;IPPK	rs41305489	CD59	-3.1	-4.4	RBP motif+ribosnitch+RBP binding+miRNA target
10	101582832	G/A	exonic	POLL	rs41291484	NF1	-2.8	-4.1	-
10	7806648	C/T	intronic	ATP5C1	rs7902363	TAF1	-4.1	-4.8	-
10	79386308	T/C	UTR3	ZCCHC24	rs147555076	BCL2	3.5	2.2	-
11	64643506	G/A	intronic	NRXN2	rs71579882	STAT3	-3.4	-2.7	-
11	72756232	C/T	intronic	STARD10	rs148235301	CREBBP	2.7	1.4	-
12	132553706	C/A	intronic	FBRSL1	rs1574157	DNMT1	5.4	3.8	-
12	45920243	T/C	UTR3	SCAF11	rs149034318	ZBTB1	-2.7	-4.0	-
12	49100521	C/T	intronic	LMBR1L	rs138195319	KLF2	-3	-4.3	-
12	53513226	C/T	UTR3	ATF7	rs73103977	SRF	-5.3	-3.1	-

12	55703100	C/A	exonic	ITGA7	rs17854601	DNMT1	5.4	3.8	-
12	57095374	C/T	UTR3	NAB2	rs3024983	DNMT1	5.4	3.8	-
12	7487993	C/T	exonic	CD163	rs7980201	JUN	-3.2	-4.5	-
12	84861228	T/C	UTR3	SLC6A15	rs143168309	RXRA	-4.2	-5.5	-
12	8917692	C/T	exonic	PHC1	rs201332046	KAT2B	6.8	2.7	ribosnitch+RBP binding
13	28387272	T/A	UTR3	FLT1	rs144398423	HOXA1	-3.8	-5.1	-
14	103742847	G/A	intronic	PPP1R13B	rs117222583	HDAC1	-4.7	-3.5	-
14	77028141	G/C	UTR5	IRF2BPL	rs76980172	ZBTB7A	8.7	3.7	-
15	25723943	G/T	exonic	ATP10A	rs17116056	GATA2	-3.5	-4.8	-
15	75346878	G/A	upstream	NEIL1	rs5745902	DNMT1	5.4	3.8	-
15	90947604	C/T	intronic	UNC45A	rs144002184	ZBTB7A	8.7	3.7	-
16	56473131	C/T	intronic	OGFOD1	rs8044359	NF1	-2.8	-4.1	-
16	6904880	C/T	intronic	RBFOX1	rs116525334	IRF1	-4.3	-5.6	-
16	75170600	A/G	UTR3	ZFP1	rs111649571	IRF5	-2.6	-3.2	RBP motif+ribosnitch+RBP binding
16	85093632	T/C	UTR3	KIAA0513	rs59156608	CTBP1	4.9	3.6	-
16	8859173	C/T	exonic	CARHSP1	rs2231706	BCL2	3.5	2.2	ribosnitch+RBP binding
17	1773615	C/T	intronic	SERPINF1	rs1894286	SOX2	10.8	5.3	-
17	3636541	T/C	UTR5	CTNS	rs111977802	REST	-2.7	-4.0	ribosnitch+RBP binding
17	39656264	T/C	intronic	STARD3	rs2934958	DNMT1	5.4	3.8	-
17	80883900	G/A	exonic	RPTOR	rs61753566	STAT5A	-3.9	-5.2	-
18	48859991	G/T	UTR3	CTIF	rs141179242	DNMT1	5.4	3.8	-
18	76960785	C/T	exonic	ZNF236	rs72987221	MAF	-3.1	-4.4	-
19	1376842	C/T	UTR3	MUM1	rs139291622	HDAC1	-4.7	-3.5	ribosnitch+RBP binding
19	19228476	G/A	exonic	NCAN	rs10406347	DNMT1	5.4	3.8	-
19	35344719	G/A	intronic	CD22	rs3746251	GATA3	-3.1	-4.4	-
19	40800745	C/T	exonic	EGLN2	rs61750953	DNMT1	5.4	3.8	ribosnitch+RBP binding
19	49711567	A/G	intronic	CPT1C	rs11881828	HDAC1	-4.7	-3.5	-
20	3875857	T/A	UTR3	MAVS	rs867335	STAT6	-4.9	-6.2	-
20	58669340	G/A	exonic	STX16	rs41276950	KLF2	-3	-4.3	ribosnitch+RBP binding
21	33915661	C/T	intronic	ATP5O	rs9979313	BCL11A	8	6.7	RBP motif+ribosnitch+RBP binding
22	23581301	G/A	upstream	IGLL1	rs131423	DNMT1	5.4	3.8	-

22	26452361	G/C	UTR3	HPS4	rs143369347	ZBTB1	-2.7	-4.0	-
22	39375109	G/A	intronic	SYNGR1	rs80181382	NANOG	2.9	1.6	-
22	40401643	T/C	exonic	SGSM3	rs9611338	SETDB1	-5.9	-7.2	-
14	105209609	G/A	UTR3	BRF1	rs118016083	KLF2	-3	-4.3	-
9	13105860	G/A	UTR3	MPDZ	rs56112316	MLLT1	-4.4	-5.7	-
3	52539085	G/A	intronic	SMIM4	rs60823713	KLF2	-3	-4.3	-
4	533446	C/T	intronic	PIGG	rs71602468	HOXA1	-3.8	-5.1	ribosnitch+RBP binding
11	78214662	C/G	UTR3	USP35	rs1055250	STAT1	-4.4	-5.7	-
12	40791017	T/A	intronic	CNTN1	rs11178389	GATA2	-3.5	-4.8	-
20	8650297	A/C	intronic	PLCB1	rs7264711	TRIM28	-4.3	-5.6	-
2	15429252	T/C	intronic	NBAS	rs11675921	HDAC1	-4.7	-3.5	-
3	159430298	A/G	intronic	IQCJ-SCHIP1; SCHIP1	rs7639707	SETDB1	-5.9	-7.2	-
4	163844366	T/C	intronic	43891	rs7670349	USF2	3.7	2.4	-
7	105193970	T/A	intronic	SRPK2	rs10277120	HEY1	5.8	4.5	-
8	10369718	A/G	intronic	MSRA	rs6984231	ZEB1	4.9	3.6	-
17	50963983	T/C	UTR3	SPAG9	rs145817549	BCL11A	8	6.7	-
10	3107210	A/G	intronic	PFKP	rs10903967	DNMT1	5.4	3.8	-
17	17216935	C/G	intronic	FLCN	rs41371953	NA	NA	NA	-
6	132459904	G/A	UTR3	STX7	rs1856352	NA	NA	NA	-
1	15518223	G/A	exonic	CASP9	rs2308941	NA	NA	NA	RBP motif+ribosnitch+RBP binding
1	156912965	C/G	exonic	PEAR1	rs41299597	NA	NA	NA	-
1	16395302	C/T	UTR3	SZRD1	rs138678090	hsa-miR-301a-p	-2.7	-5.1	ribosnitch+RBP binding
1	164850507	T/C	UTR3	PBX1	rs149358308	NA	NA	NA	RBP motif+ribosnitch+RBP binding+miRNA target
1	16946949	G/A	exonic	CROCC	rs7545185	NA	NA	NA	ribosnitch+RBP binding
1	208210194	A/C	intronic	PLXNA2	rs114080113	NA	NA	NA	-
1	228394658	C/A	UTR3	TRIM11	rs141363391	NA	NA	NA	-
1	248849323	C/A	UTR3	ZNF672	rs12045382	NA	NA	NA	ribosnitch+RBP binding
1	25228228	G/A	exonic	SYF2	rs35324907	NA	NA	NA	-
1	47218494	T/C	UTR3	TAL1	rs1010812	NA	NA	NA	-
1	87346555	G/A	UTR3	LMO4	rs12030163	NA	NA	NA	-
3	149153745	A/G	intronic	HPS3	rs78083599	NA	NA	NA	-
3	15251321	C/T	UTR3	CAPN7	rs111665976	NA	NA	NA	ribosnitch+RBP binding
3	50184639	T/C	exonic	SEMA3F		NA	NA	NA	-

3	50363617	C/G	UTR3	CACNA2D2		NA	NA	NA	-
4	186212115	G/T	UTR3	CYP4V2	rs77175303	NA	NA	NA	-
4	82818908	G/A	UTR3	SEC31A	rs115684235	NA	NA	NA	-
4	82926063	A/G	UTR3	LIN54	rs77238199	NA	NA	NA	-
5	66182181	A/T	UTR3	SREK1	rs187444452	NA	NA	NA	-
6	26393739	A/C	UTR3	BTN2A2	rs77055120	NA	NA	NA	ribosnitch+RBP binding
7	19719579	T/G	UTR3	TMEM196	rs78430922	NA	NA	NA	-
7	76286756	G/A	UTR3	SRRM3	rs1859790	NA	NA	NA	-
8	143298141	G/A	UTR3	ZNF696	rs71518788	NA	NA	NA	-
9	109378354	T/C	UTR3	PTPN3	rs139439518	NA	NA	NA	-
9	15468045	A/G	intronic	PSIP1		NA	NA	NA	-
9	89012527	C/G	UTR3	SHC3	rs2316282	NA	NA	NA	-
10	1717353	C/A	intronic	ADARB2	rs11250759	NA	NA	NA	-
10	30848638	C/T	exonic	ZNF438	rs41289001	NA	NA	NA	-
11	13388687	G/C	UTR3	BTBD10		NA	NA	NA	-
11	95845106	C/T	exonic	MTMR2	rs113897932	NA	NA	NA	-
12	117454597	T/C	UTR3	KSR2	rs181234334	NA	NA	NA	-
12	14500449	T/C	UTR3	ATF7IP	rs150504302	NA	NA	NA	-
12	27802919	G/A	UTR3	KLHL42	rs7294360	NA	NA	NA	-
12	56645296	C/T	exonic	ATP5B	rs41291993	NA	NA	NA	-
12	7485173	G/A	exonic	CD163	rs61729512	NA	NA	NA	-
13	32274849	A/G	exonic	FRY	rs199709966	NA	NA	NA	ribosnitch+RBP binding
13	51367807	T/C	exonic	INTS6	rs61749884	NA	NA	NA	-
13	83878283	A/G	UTR3	SLITRK1	rs3737193	NA	NA	NA	-
14	47560524	T/C	intronic	MDGA2		NA	NA	NA	-
16	583988	G/A	UTR3	PIGQ	rs80239021	NA	NA	NA	ribosnitch+RBP binding
16	88652556	C/G	exonic	MVD	.	NA	NA	NA	-
16	88716447	G/A	exonic	PIEZO1	rs35917730	NA	NA	NA	ribosnitch+RBP binding
17	50551730	C/G	intronic	SPATA20	rs145236385	NA	NA	NA	-
17	510449	C/A	UTR3	VPS53	rs28637278	NA	NA	NA	-
17	78172139	G/A	UTR3	SYNGR2	rs535896260	NA	NA	NA	-
17	81994824	G/A	exonic	ASPSCR1	rs116149334	NA	NA	NA	ribosnitch+RBP binding
20	44907588	C/G	UTR3	YWHAB	rs188983062	hsa-miR-301a-p	-1.3	-4.2	-
21	33551922	G/A	exonic	SON	rs61746013	NA	NA	NA	-
22	46353091	G/A	intronic	TRMU	rs80255607	NA	NA	NA	-
1	108932814	T/C	UTR3	GPSM2	rs115590945	NA	NA	NA	-

13	113092088	A/G	intronic	MCF2L	rs80238657	NA	NA	NA	-
15	22839090	C/T	intronic	NIPA2		NA	NA	NA	-
22	23315819	G/A	UTR3	BCR	rs1042660	NA	NA	NA	-
6	31356259	T/A	exonic	HLA-B	rs151341293	NA	NA	NA	ribosnitch+RBP binding
4	44407356	G/C	intronic	KCTD8	rs73247543	NA	NA	NA	-
19	44893972	G/A	exonic	TOMM40	rs1160983	NA	NA	NA	-
20	46043241	C/T	exonic	SLC12A5	rs35804246	NA	NA	NA	-
2	48490331	G/T	intronic	PPP1R21	rs77137673	NA	NA	NA	-
19	6680499	C/T	intronic	C3	rs11569557	NA	NA	NA	-
10	82274230	A/G	intronic	NRG3	rs7096123	NA	NA	NA	-
16	25838628	C/T	intronic	HS3ST4	rs4511540	NA	NA	NA	-
3	183762407	T/C	intronic	YEATS2	rs262990	NA	NA	NA	-
4	153731082	T/C	intronic	RNF175	rs2579918	NA	NA	NA	-
4	164365248	C/A	intronic	MARCH	rs924907	NA	NA	NA	-
4	15607054	A/G	intronic	FBXL5	rs75836515	NA	NA	NA	-

NA: Not available, Fold changes are extracted from the following GEO Id: AD (GSE1297,GSE118553,GSE132903,GSE110298,GSE84422,GSE33000), PD (GSE7621,GSE20168,GSE20292)

Table S6: Count table for significant differentially expressed genes in AD and PD

(Supplementary Table S6.xlsx)

Table S7: Comparison of BA9 gene expression (disease) with other RNA seq data (AD/PD)

GEO	Description
GSE157827	Single-nucleus transcriptome analysis of 169,496 nuclei from the prefrontal cortical samples of AD patients and normal control (NC) subjects
GSE125583	RNA from fusiform gyrus of Alzheimer's or Neurologically Normal post-mortem tissue
GSE130752	RNA seq human SVZ of control and Parkinson disease donors
GSE114517	RNA-seq of substantia nigra pars compacta from Parkinson's disease patients
GSE1297	Micro array data from hippocampus CA1

Log2FC (Padj <0.05) Single-nucleus transcriptome analysis of 169,496 nuclei from the prefrontal cortical samples of AD patients and normal control (NC) subjects (GSE157827)						
Gene name	Astrocytes	Endothelial cell	Inhibitory neurons	Microglia	Oligodendrocytes	Bulk-RNA seq (BA9)
COL5A3	1.95	0.00	0.00	0.00	0.00	2.93
TRPS1	1.47	0.00	0.00	0.00	0.00	2.63
SASH1	1.22	0.52	0.00	0.00	0.00	3.34
PBXIP1	1.04	0.00	0.00	0.00	0.00	3.88
MT1G	1.01	0.00	0.00	0.00	0.00	2.84
ZBTB20	1.00	0.00	0.00	0.00	0.81	3.43
BCL6	0.99	0.00	0.00	0.00	0.00	2.47
EPHX1	0.90	0.00	0.00	0.00	0.00	3.70
NFIB	0.83	0.00	0.00	0.00	0.00	2.14
KANK1	0.68	0.00	0.00	0.00	0.89	3.10
ZHX2	0.63	0.00	0.00	0.00	0.61	2.96
LRP4	0.62	0.00	0.00	0.00	0.00	4.97
MID1	0.61	0.00	0.00	0.00	0.00	3.22
DOCK4	0.57	0.00	0.00	1.72	0.00	9.47
IL17RB	0.55	0.00	0.00	0.00	0.00	4.87
FLT1	0.00	3.10	0.00	0.00	0.00	5.25
SPARC	0.00	1.55	0.00	0.00	0.00	3.61
TGM2	0.00	1.40	0.00	0.00	0.00	2.07
TNS1	0.00	1.36	0.00	0.00	0.00	3.18
RGCC	0.00	0.52	0.00	0.00	0.98	5.23
KTN1	0.00	0.57	0.00	0.00	0.00	2.18
SHC2	0.00	0.55	0.00	0.00	0.00	3.27
KAZN	0.00	0.00	1.12	0.00	0.00	2.34
BHLHE41	0.00	0.00	0.00	1.20	0.00	3.56
VASH1	0.00	0.00	0.00	0.96	0.00	2.49
FCHSD2	0.00	0.00	0.00	0.88	0.89	2.73
CHD9	0.00	0.00	0.00	0.65	0.00	2.87
TCF12	0.00	0.00	0.00	0.62	1.33	2.78
DIP2A	0.00	0.00	0.00	0.63	0.00	4.27
ITSN2	0.00	0.00	0.00	0.61	0.00	5.47
BIN1	0.00	0.00	0.00	0.57	0.98	2.40
PHF20	0.00	0.00	0.00	0.55	0.00	3.67
TF	0.00	0.00	0.00	0.00	2.13	3.11
MAN2A1	0.00	0.00	0.00	0.00	1.96	2.22
CLMN	0.00	0.00	0.00	0.00	1.84	2.46
PHLPP1	0.00	0.00	0.00	0.00	1.79	3.03
CRYAB	0.00	0.00	0.00	0.00	1.75	9.73
FRYL	0.00	0.00	0.00	0.00	1.74	6.15
ABCA8	0.00	0.00	0.00	0.00	1.66	2.29
QKI	0.00	0.00	0.00	0.00	1.62	3.10
SELENOP	0.00	0.00	0.00	0.00	1.34	6.11
GPM6B	0.00	0.00	0.00	0.00	1.34	5.33
CDH19	0.00	0.00	0.00	0.00	1.32	2.94
SUN2	0.00	0.00	0.00	0.00	1.29	3.27

FA2H	0.00	0.00	0.00	0.00	1.28	2.47
CCP110	0.00	0.00	0.00	0.00	1.24	3.02
SLCO3A1	0.00	0.00	0.00	0.00	1.23	2.32
EFHD1	0.00	0.00	0.00	0.00	1.18	2.40
KCNMB4	0.00	0.00	0.00	0.00	1.13	2.37
LRRC1	0.00	0.00	0.00	0.00	1.06	2.61
CPQ	0.00	0.00	0.00	0.00	0.97	6.38
DIP2C	0.00	0.00	0.00	0.00	0.83	3.76
KCNJ2	0.00	0.00	0.00	0.00	0.82	5.24
MID1IP1	0.00	0.00	0.00	0.00	0.71	2.55
PPM1B	0.00	0.00	0.00	0.00	0.71	2.60
SGMS1	0.00	0.00	0.00	0.00	0.69	4.75
TJAP1	0.00	0.00	0.00	0.00	0.65	5.85
ANKRD28	0.00	0.00	0.00	0.00	0.64	4.63
CDKN1C	0.00	0.00	0.00	0.00	0.64	3.06
SIRT2	0.00	0.00	0.00	0.00	0.62	2.62
RALGDS	0.00	0.00	0.00	0.00	0.56	2.48
PACS2	0.00	0.00	0.00	0.00	0.54	2.76
MAPK1	0.00	0.60	0.00	0.50	0.00	3.90
FGFR1	0.80	0.00	0.00	0.00	0.00	3.63
PSMD1	0.00	-0.50	0.00	-0.80	0.00	-3.50
SIRT1	0.00	1.25	0.00	0.00	0.00	2.80
TOMM22	0.00	0.00	0.00	0.00	-0.50	-2.93
TIMM10	0.00	0.00	0.00	0.00	0.00	-2.23
PGK1	-0.50	0.00	0.00	-0.50	0.00	-3.68

Log2FC (Padj <0.05) single cell RNA from fusiform gyrus of Alzheimer's or Neurologically Normal post-mortem tissue (GSE125583)			Log2FC (Padj <0.05) RNA seq human SVZ of control and Parkinson disease donors (GSE130752)			Log2FC (Padj <0.05) RNA-seq of substantia nigra pars compacta from Parkinson's disease patients (GSE114517)		
Gene name	Microglia	Bulk-RNA seq (BA9)	Gene name	SVZ RNA seq	Bulk-RNA seq (BA9)	Gene name	SNc-GSE114517	Bulk-RNA seq (BA9)
CDKN1C	1.8	3.1	*TBC1D9*	-1.33	-3.56	TUB	-0.93	-2.73
CPQ	0.9	6.4	*EFHD1*	4.54	2.40	IGFBP5	1.29	2.64
SIRT2	0.5	2.6	*SUN2*	1.58	3.27	TNS1	0.74	3.18
KANK1	2.3	3.1	*SLCO3A1*	2.17	2.32	BRINP1	0.92	5.33
KIAA0930	0.9	5.3	*KCNMB4*	2.51	2.37	RALGDS	0.69	2.48
SASH1	5.0	3.3	*LHPP*	2.60	2.31	PLCH1	-1.38	-2.60
SUN2	1.8	3.3	*SPARC*	2.76	3.61	KCNJ2	0.80	5.24
SLCO3A1	7.2	2.3	*PHLPP1*	2.95	3.03	LRRC1	0.94	2.61
RGCC	1.7	5.2	*CLMN*	3.07	2.46			
COL5A3	2.8	2.9	*SIRT2*	3.10	2.62			
PPM1B	0.4	2.6	*SASH1*	3.28	3.34			
MAPK1	0.6	3.9	*GPM6B*	3.69	5.33			
PBXIP1	0.9	3.9	*CHST3*	3.86	2.74			
MID1IP1	1.2	2.5	*CRYAB*	4.16	9.73			
BCL6	1.2	2.5	*KAZN*	4.20	2.34			
LHPP	1.4	2.3	*KANK1*	4.29	3.10			
SPARC	7.6	3.6	*COL5A3*	4.70	2.93			
TGM2	4.0	2.1	*FA2H*	4.71	2.47			
TNS1	3.8	3.2	*CDH19*	4.89	2.94			
TRPS1	1.2	2.6	*ABCA8*	5.26	2.29			
ZBTB16	2.3	2.7	*TF*	5.61	3.11			
CLMN	7.3	2.5						
SAFB2	0.4	4.3						
RANBP9	-1.1	-2.7						
POLR3C	-0.2	-6.6						
RBFOX2	-4.4	-3.2						
ARIH1	-0.3	-6.6						
GLS	-1.7	-3.6						
GSS	-0.3	-2.8						
XIAP	-0.4	-2.9						
MYH10	-1.1	-7.0						
PAFAH1B1	-0.3	-2.4						
GOLT1B	-0.4	-4.0						
ATP6V1C1	-0.4	-2.5						
DNAJC10	-1.3	-5.8						
SLC25A36	-1.6	-3.9						
GUF1	-0.4	-3.0						
TUB	-1.8	-2.7						
WDR77	-0.5	-3.5						
TMEM185B	-0.4	-3.6						
RNMT	-0.7	-2.6						
ZMYM4	-0.6	-4.0						

SCAMP1	-0.9	-2.5
RABGAP1L	-0.6	-2.8

Log2FC (Padj <0.05) Micro array data fromhippocampus CA1 (GSE1297)		
Gene name	Bulk-RNA seq (BA9)	Hippocampus CA1 (AD)
RBM6	4.62	3.6
STAG2	2.09	2.6
TOB2	8.77	5.3
CRY1	5.28	2.9
TMCC1	2.41	8.5
KDM4C	4.62	7
KDSR	3.04	5.7
PNISR	2.76	5.3
KAT2A	3.27	2.7
MSX1	2.53	3
LUC7L3	2.42	4.9
ATP8B1	2.45	4.2
POU2F1	2.51	4.6
VPS13C	3.27	2.6
C2orf42	3.28	6.8
ZNF692	2.17	2.7
SCAF4	4.02	2.6
FBXW4	2.56	7.2
MZF1	2.39	3.2
ZNF175	2.32	5.6
ALMS1	2.90	3.2
NAA16	6.17	3.5
KATNBL1	8.43	8.4
ZNF34	2.35	3
PABPN1	2.90	5
ZNF611	2.16	3
RBM48	3.89	5
ZNF160	2.35	3.9
RUBCN	2.33	4.1
SAE1	-3.24	-3
ACTR2	-2.16	-5.6
ATP6AP2	-2.18	-3.2
OLFM1	-2.53	-2.6
B3GNT2	-2.44	-3.3
GNB5	-3.34	-4.8
AFG3L2	-3.11	-4.3
CKAP4	-2.82	-6.3
CSNK1D	-2.24	-6.7
CD55	-2.63	-5.3
TOR1A	-3.47	-7.3
ENSA	-2.00	-3.2
ALAS1	-2.27	-3.7
SHANK2	-3.78	-3
NNT	-2.36	-5.5

OPN3	-6.48	-2.6
MTCH2	-2.47	-7.1
FUCA1	-2.17	-2.7
MOB4	-2.76	-3.2
TRAF3IP1	-2.75	-2.9
FBXO9	-2.61	-4.1
GBE1	-2.20	-2.9
GHITM	-2.36	-6.1
TMEM97	-7.21	-3.9
MCAT	-5.53	-4.1
GNB1	-2.14	-6.3
BZW2	-3.26	-6.5
NDUFAF4	-3.76	-3.4
SLC25A6	-2.83	-7.4
APLP2	-6.54	-5.6
ILF3	-3.59	-5.1
INPP4A	-2.17	-3.5
ARHGDIG	-6.43	-3
STS	-2.67	-2.5
MDH2	-3.17	-5.8
AFDN	-2.99	-3.6
NDUFA9	-4.21	-6.6
OSBP	-5.73	-5.5
UTP11	-3.35	-3.2
UCHL5	-6.47	-4.1
RAB14	-3.16	-2.5
ATP6V1A	-8.01	-2.5
C20orf27	-3.99	-3.4
ELP3	-2.80	-5.8
ATXN7L3B	-3.77	-5.5
BRF2	-3.60	-3.7
SLC39A9	-3.28	-3
HMCES	-3.65	-5
SMYD2	-3.89	-3.3
RAB2A	-3.87	-6.9
RBM3	-3.69	-6.6
SLC18A2	-2.97	-4
STRN	-2.91	-4
TBCC	-3.03	-4.6
TPBG	-4.25	-3.5
TPI1	-2.95	-2.9
UBE2B	-3.22	-2.9
UBE2N	-3.12	-5.3
UMPS	-2.62	-6.7
ARPC5L	-2.99	-2.6
AGPS	-4.38	-7.4
BECN1	-2.69	-6.7
CDS2	-2.96	-2.8
ACVR1B	-3.15	-2.8
FIBP	-4.09	-4.3

PTDSS1	-2.87	-3.4
TOMM70	-3.24	-7.9
G3BP2	-3.06	-6.2
MFN2	-3.24	-3.5

Table S8: Tissue specific Functional interaction between TFs and DEG						
source	Source exp	source type	target	target_exp	target type	edge
NF1	DOWN	Reported TF	STAT6	DOWN	Reported TF	complex input
ALMS1	UP	Reported DG	PAFAH1B1	DOWN	Reported DG	complex input
NF1	DOWN	Reported TF	STAT1	DOWN	Reported TF	complex input
NF1	DOWN	Reported TF	STAT3	DOWN	Reported TF	complex input
HDAC1	DOWN	Reported TF	WDR77	DOWN	Reported DG	complex input
PSMD1	DOWN	Reported DG	UCHL5	DOWN	Reported DG	complex input
CSNK1D	DOWN	Reported DG	PAFAH1B1	DOWN	Reported DG	complex input
KAT2A	UP	Reported DG	KAT2B	UP	Reported TF	complex input
CREBBP	UP	Reported TF	KAT2B	UP	Reported TF	complex input
CREBBP	UP	Reported TF	KAT2A	UP	Reported DG	complex input
CCP110	UP	Reported DG	PAFAH1B1	DOWN	Reported DG	complex input
ACTR2	DOWN	Reported DG	AFDN	DOWN	Reported DG	complex input
CTBP1	UP	Reported TF	ZEB1	UP	Reported TF	complex input
KAT2B	UP	Reported TF	SIRT1	UP	Reported DG	complex input
ALMS1	UP	Reported DG	CCP110	UP	Reported DG	complex input
GATA3	DOWN	Reported TF	TAL1	DOWN	Reported variant/TF	complex input
CHD9	UP	Reported DG	NFYA	DOWN	Reported TF	complex input
ALMS1	UP	Reported DG	CSNK1D	DOWN	Reported DG	complex input
GATA2	DOWN	Reported TF	TAL1	DOWN	Reported variant/TF	complex input
ACTR2	DOWN	Reported DG	BIN1	UP	Reported DG	complex input
NDUFA9	DOWN	Reported DG	NDUFAF4	DOWN	Reported DG	complex input
GATA2	DOWN	Reported TF	JUN	DOWN	Reported TF	complex input

HDAC1	DOWN	Reported TF	REST	DOWN	Reported TF	complex input
HDAC1	DOWN	Reported TF	NCOR1	DOWN	Reported TF	complex input
DNMT1	UP	Reported TF	HDAC1	DOWN	Reported TF	complex input
NCOR1	DOWN	Reported TF	NR2C1	UP	Reported TF	complex input
PAFAH1B1	DOWN	Reported DG	STAG2	UP	Reported DG	complex input
BIN1	UP	Reported DG	TF	UP	Reported DG	complex input
NF1	DOWN	Reported TF	STAT5A	DOWN	Reported TF	complex input
GNB1	DOWN	Reported DG	TGM2	UP	Reported DG	complex input
DNMT1	UP	Reported TF	E2F1	DOWN	Reported TF	complex input
NF1	DOWN	Reported TF	POU2F1	UP	Reported DG	complex input
CRY1	UP	Reported DG	CSNK1D	DOWN	Reported DG	complex input
ACTR2	DOWN	Reported DG	TF	UP	Reported DG	complex input
GATA3	DOWN	Reported TF	POU2F1	UP	Reported DG	complex input
ATP6V1A	DOWN	Reported DG	ATP6V1C1	DOWN	Reported DG	complex input
TOMM22	DOWN	Reported DG	TOMM70	DOWN	Reported DG	complex input
GNB1	DOWN	Reported DG	TUB	DOWN	Reported DG	complex input
BRF2	DOWN	Reported DG	POLR3C	DOWN	Reported DG	complex input
CCP110	UP	Reported DG	CSNK1D	DOWN	Reported DG	complex input
CREBBP	UP	Reported TF	SMARCA1	UP	Reported TF	complex input
CHD9	UP	Reported DG	CREBBP	UP	Reported TF	complex input
JUN	DOWN	Reported TF	POU2F1	UP	Reported DG	complex input
SLC25A6	DOWN	Reported DG	TIMM10	DOWN	Reported DG	complex input
GNB1	DOWN	Reported DG	KCNJ2	UP	Reported DG	complex input
CREBBP	UP	Reported TF	POU2F1	UP	Reported DG	complex input
STAG2	UP	Reported DG	SUN2	UP	Reported DG	complex input

BRF2	DOWN	Reported DG	POU2F1	UP	Reported DG	complex input
NFYA	DOWN	Reported TF	RXRA	DOWN	Reported TF	complex input
POLR3C	DOWN	Reported DG	POU2F1	UP	Reported DG	complex input
STAT1	DOWN	Reported TF	STAT5A	DOWN	Reported TF	Binding
STAT1	DOWN	Reported TF	STAT6	DOWN	Reported TF	Binding
STAT5A	DOWN	Reported TF	STAT6	DOWN	Reported TF	Binding
STAT2	DOWN	Reported TF	STAT5A	DOWN	Reported TF	Binding
NANOG	UP	Reported TF	STAT3	DOWN	Reported TF	Binding
STAT3	DOWN	Reported TF	STAT6	DOWN	Reported TF	Binding
STAT2	DOWN	Reported TF	STAT6	DOWN	Reported TF	Binding
STAT3	DOWN	Reported TF	STAT5A	DOWN	Reported TF	Binding
CHD9	UP	Reported DG	NFYA	DOWN	Reported TF	Reaction
CHD9	UP	Reported DG	CREBBP	UP	Reported TF	Reaction
CREBBP	UP	Reported TF	POU2F1	UP	Reported DG	Reaction
NFYA	DOWN	Reported TF	RXRA	DOWN	Reported TF	Reaction
RXRA	DOWN	Reported TF	CREBBP	UP	Reported TF	Inhibit
RXRA	DOWN	Reported TF	CHD9	UP	Reported DG	Inhibit
E2F1	DOWN	Reported TF	STAT3	DOWN	Reported TF	Inhibit
HDAC1	DOWN	Reported TF	IRF1	DOWN	Reported TF	Inhibit
HDAC1	DOWN	Reported TF	SIRT1	UP	Reported DG	Inhibit
CREBBP	UP	Reported TF	NFIB	UP	Reported DG	activate
JUN	DOWN	Reported TF	NFIB	UP	Reported DG	activate
CREBBP	UP	Reported TF	CTBP1	UP	Reported TF	activate
E2F1	DOWN	Reported TF	SIRT1	UP	Reported DG	activate
NCOR1	DOWN	Reported TF	TRIM28	DOWN	Reported TF	activate

CHD9	UP	Reported DG	NCOR1	DOWN	Reported TF	activate
GATA3	DOWN	Reported TF	HDAC1	DOWN	Reported TF	activate
E2F1	DOWN	Reported TF	HDAC1	DOWN	Reported TF	activate
NFIB	UP	Reported DG	CREBBP	UP	Reported TF	Inhibit
NCOR1	DOWN	Reported TF	CREBBP	UP	Reported TF	Inhibit
NFIB	UP	Reported DG	JUN	DOWN	Reported TF	Inhibit
CTBP1	UP	Reported TF	CREBBP	UP	Reported TF	Inhibit
SIRT1	UP	Reported DG	E2F1	DOWN	Reported TF	Inhibit
TRIM28	DOWN	Reported TF	NCOR1	DOWN	Reported TF	Inhibit
NCOR1	DOWN	Reported TF	CHD9	UP	Reported DG	Inhibit
JUN	DOWN	Reported TF	CREBBP	UP	Reported TF	Inhibit
HDAC1	DOWN	Reported TF	GATA3	DOWN	Reported TF	Inhibit
HDAC1	DOWN	Reported TF	E2F1	DOWN	Reported TF	Inhibit
PSMD1	DOWN	Reported DG	NF1	DOWN	Reported TF	activate
JUN	DOWN	Reported TF	ATP8B1	UP	Reported DG	activate
TCF12	UP	Reported DG/TF	MSX1	UP	Reported DG	activate
STAT3	DOWN	Reported TF	PGK1	DOWN	Reported DG	activate
SRF	DOWN	Reported TF	MAPK1	UP	Reported DG	activate
STAT5A	DOWN	Reported TF	BCL2	UP	Reported TF	activate
ZBTB7A	UP	Reported TF	MAPK1	UP	Reported DG	activate
CSNK1D	DOWN	Reported DG	CD59	DOWN	Reported TF	activate
STAT1	DOWN	Reported TF	RNMT	DOWN	Reported DG	activate
JUN	DOWN	Reported TF	GATA3	DOWN	Reported TF	activate
ZBTB7A	UP	Reported TF	GATA2	DOWN	Reported TF	activate
TRIM28	DOWN	Reported TF	MAPK1	UP	Reported DG	activate

RXRA	DOWN	Reported TF	HDAC1	DOWN	Reported TF	activate
SRF	DOWN	Reported TF	GATA3	DOWN	Reported TF	activate
MEF2A	UP	Reported TF	GATA3	DOWN	Reported TF	activate
STAT5A	DOWN	Reported TF	IRF1	DOWN	Reported TF	activate
USF2	UP	Reported TF	SIRT1	UP	Reported DG	activate
TAF1	DOWN	Reported TF	STAT5A	DOWN	Reported TF	activate
STAT6	DOWN	Reported TF	GATA3	DOWN	Reported TF	activate
STAT3	DOWN	Reported TF	GATA3	DOWN	Reported TF	activate
ZBTB7A	UP	Reported TF	KAT2B	UP	Reported TF	activate
USF2	UP	Reported TF	STAT5A	DOWN	Reported TF	activate
STAT6	DOWN	Reported TF	IRF1	DOWN	Reported TF	activate
STAT2	DOWN	Reported TF	IRF1	DOWN	Reported TF	activate
STAT3	DOWN	Reported TF	IRF1	DOWN	Reported TF	activate
MEF2A	UP	Reported TF	KLF2	DOWN	Reported TF	activate
TAL1	DOWN	Reported variant/TF	MAPK1	UP	Reported DG	activate
TAL1	DOWN	Reported variant/TF	SELENOP	UP	Reported DG	activate
SRF	DOWN	Reported TF	FGFR1	UP	Reported DG	activate
TCF12	UP	Reported DG/TF	PGK1	DOWN	Reported DG	activate
TRIM28	DOWN	Reported TF	HDAC1	DOWN	Reported TF	activate
TAF1	DOWN	Reported TF	MAPK1	UP	Reported DG	activate
TAL1	DOWN	Reported variant/TF	STAT5A	DOWN	Reported TF	activate
SRF	DOWN	Reported TF	HEY1	UP	Reported TF	activate
MAPK1	UP	Reported DG	BCL2	UP	Reported TF	activate

STAT5A	DOWN	Reported TF	GATA3	DOWN	Reported TF	activate
STAT3	DOWN	Reported TF	STAT2	DOWN	Reported TF	activate
JUN	DOWN	Reported TF	BCL2	UP	Reported TF	activate
GATA3	DOWN	Reported TF	CD55	DOWN	Reported DG	activate
TCF12	UP	Reported DG/TF	HOXA1	DOWN	Reported TF	activate
REST	DOWN	Reported TF	BIN1	UP	Reported DG	activate
TCF12	UP	Reported DG/TF	GATA3	DOWN	Reported TF	activate
STAT6	DOWN	Reported TF	BCL2	UP	Reported TF	activate
STAT3	DOWN	Reported TF	BCL2	UP	Reported TF	activate
STAT2	DOWN	Reported TF	BCL2	UP	Reported TF	activate
STAT1	DOWN	Reported TF	BCL2	UP	Reported TF	activate
TAL1	DOWN	Reported variant/TF	FGFR1	UP	Reported DG	activate
SOX2	UP	Reported TF	NANOG	UP	Reported TF	activate
CREBBP	UP	Reported TF	BCL2	UP	Reported TF	activate
TAF1	DOWN	Reported TF	STAT1	DOWN	Reported TF	activate
STAT1	DOWN	Reported TF	BCL6	UP	Reported DG	activate
MEF2A	UP	Reported TF	JUN	DOWN	Reported TF	activate
STAT3	DOWN	Reported TF	BCL6	UP	Reported DG	activate
STAT5A	DOWN	Reported TF	RNMT	DOWN	Reported DG	activate
STAT1	DOWN	Reported TF	JUN	DOWN	Reported TF	activate
STAT3	DOWN	Reported TF	JUN	DOWN	Reported TF	activate
HDAC1	DOWN	Reported TF	KAT2B	UP	Reported TF	Inhibit
NCOR1	DOWN	Reported TF	SIRT1	UP	Reported DG	Inhibit
NCOR1	DOWN	Reported TF	RXRA	DOWN	Reported TF	Inhibit

HDAC1	DOWN	Reported TF	JUN	DOWN	Reported TF	Inhibit
JUN	DOWN	Reported TF	RXRA	DOWN	Reported TF	Inhibit
BCL6	UP	Reported DG	STAT6	DOWN	Reported TF	Inhibit
NFIB	UP	Reported DG	POU2F1	UP	Reported DG	Inhibit
SIRT1	UP	Reported DG	NCOR1	DOWN	Reported TF	activate
RXRA	DOWN	Reported TF	NCOR1	DOWN	Reported TF	activate
JUN	DOWN	Reported TF	HDAC1	DOWN	Reported TF	activate
RXRA	DOWN	Reported TF	JUN	DOWN	Reported TF	activate
STAT6	DOWN	Reported TF	BCL6	UP	Reported DG	activate
POU2F1	UP	Reported DG	NFIB	UP	Reported DG	activate
JUN	DOWN	Reported TF	MAPK1	UP	Reported DG	activate
FGFR1	UP	Reported DG	MAPK1	UP	Reported DG	activate
STAT1	DOWN	Reported TF	STAT2	DOWN	Reported TF	activate
MAPK1	UP	Reported DG	STAT1	DOWN	Reported TF	activate
UBE2B	DOWN	Reported DG	UBE2N	DOWN	Reported DG	activate
FBXO9	DOWN	Reported DG	FBXW4	UP	Reported DG	activate
MAPK1	UP	Reported DG	JUN	DOWN	Reported TF	activate
MAPK1	UP	Reported DG	FGFR1	UP	Reported DG	activate
STAT2	DOWN	Reported TF	STAT1	DOWN	Reported TF	activate
STAT1	DOWN	Reported TF	MAPK1	UP	Reported DG	activate
UBE2N	DOWN	Reported DG	UBE2B	DOWN	Reported DG	activate
FBXW4	UP	Reported DG	FBXO9	DOWN	Reported DG	activate
NANOG	UP	Reported TF	REST	DOWN	Reported TF	activate
GATA2	DOWN	Reported TF	MAPK1	UP	Reported DG	activate
CUX1	UP	Reported TF	STAT5A	DOWN	Reported TF	activate

FLT1	UP	Reported variant/DG	SHC2	UP	Reported DG	activate
TAF1	DOWN	Reported TF	UCHL5	DOWN	Reported DG	activate
STAT1	DOWN	Reported TF	ZNF611	UP	Reported DG	activate
TAL1	DOWN	Reported variant/TF	TF	UP	Reported DG	activate
GATA2	DOWN	Reported TF	STAT5A	DOWN	Reported TF	activate
HEY1	UP	Reported TF	ZNF175	UP	Reported DG	activate
JUN	DOWN	Reported TF	ZEB1	UP	Reported TF	activate
TAF1	DOWN	Reported TF	ZNF175	UP	Reported DG	activate
ARIH1	DOWN	Reported DG	UBE2N	DOWN	Reported DG	activate
REST	DOWN	Reported TF	TCF12	UP	Reported DG/TF	activate
STAT1	DOWN	Reported TF	STAT3	DOWN	Reported TF	activate
TAL1	DOWN	Reported variant/TF	ZEB1	UP	Reported TF	activate
STAT2	DOWN	Reported TF	ZNF34	UP	Reported DG	activate
FGFR1	UP	Reported DG	SHC2	UP	Reported DG	activate
SRF	DOWN	Reported TF	STAT5A	DOWN	Reported TF	activate
HEY1	UP	Reported TF	STAT5A	DOWN	Reported TF	activate
GATA3	DOWN	Reported TF	MAF	DOWN	Reported TF	activate
STAT1	DOWN	Reported TF	ZNF175	UP	Reported DG	activate
GATA2	DOWN	Reported TF	ZEB1	UP	Reported TF	activate
STAT1	DOWN	Reported TF	ZNF160	UP	Reported DG	activate
E2F1	DOWN	Reported TF	TRIM28	DOWN	Reported TF	activate
GNB1	DOWN	Reported DG	MAPK1	UP	Reported DG	activate
GATA2	DOWN	Reported TF	NFIB	UP	Reported DG	activate

HOXA1	DOWN	Reported TF	RXRA	DOWN	Reported TF	activate
BCL6	UP	Reported DG	JUN	DOWN	Reported TF	activate
GNB1	DOWN	Reported DG	STRN	DOWN	Reported DG	activate
STAT1	DOWN	Reported TF	ZNF34	UP	Reported DG	activate
E2F1	DOWN	Reported TF	SMYD2	DOWN	Reported DG	activate
RXRA	DOWN	Reported TF	STAT5A	DOWN	Reported TF	activate
GATA2	DOWN	Reported TF	QKI	UP	Reported DG	activate
MAPK1	UP	Reported DG	STAT3	DOWN	Reported TF	activate
IRF1	DOWN	Reported TF	STAT1	DOWN	Reported TF	activate
JUN	DOWN	Reported TF	TCF12	UP	Reported DG/TF	activate
GNB5	DOWN	Reported DG	MAPK1	UP	Reported DG	activate
GATA2	DOWN	Reported TF	TCF12	UP	Reported DG/TF	activate
CREBBP	UP	Reported TF	FLT1	UP	Reported variant/DG	activate
TAL1	DOWN	Reported variant/TF	TCF12	UP	Reported DG/TF	activate
SRF	DOWN	Reported TF	ZNF175	UP	Reported DG	activate
BCL11A	UP	Reported TF	GATA3	DOWN	Reported TF	activate
CREBBP	UP	Reported TF	TF	UP	Reported DG	activate
BCL11A	UP	Reported TF	FGFR1	UP	Reported DG	activate
STAT2	DOWN	Reported TF	ZNF611	UP	Reported DG	activate
NFIB	UP	Reported DG	POLR3C	DOWN	Reported DG	activate
HEY1	UP	Reported TF	TCF12	UP	Reported DG/TF	activate
USF2	UP	Reported TF	ZNF175	UP	Reported DG	activate
ARIH1	DOWN	Reported DG	STAT1	DOWN	Reported TF	activate
E2F1	DOWN	Reported TF	STAT5A	DOWN	Reported TF	activate

HOXA1	DOWN	Reported TF	NCOR1	DOWN	Reported TF	activate
ARIH1	DOWN	Reported DG	PPM1B	UP	Reported DG	activate
IRF1	DOWN	Reported TF	MAPK1	UP	Reported DG	activate
STAT2	DOWN	Reported TF	ZNF160	UP	Reported DG	activate
STAT1	DOWN	Reported TF	STAT3	DOWN	Reported TF	co-exp
HDAC1	DOWN	Reported TF	DNMT1	UP	Reported TF	co-exp
TRIM28	DOWN	Reported TF	TRIM28	DOWN	Reported TF	co-exp
STAT5A	DOWN	Reported TF	MAPK1	UP	Reported DG	co-exp
KAT2B	UP	Reported TF	CREBBP	UP	Reported TF	co-exp
STAT2	DOWN	Reported TF	STAT1	DOWN	Reported TF	co-exp
BECN1	DOWN	Reported DG	BCL2	UP	Reported TF	co-exp
FGFR1	UP	Reported DG	FGFR1	UP	Reported DG	co-exp
DNMT1	UP	Reported TF	SIRT1	UP	Reported DG	co-exp
MOB4	DOWN	Reported DG	STRN	DOWN	Reported DG	co-exp
UCHL5	DOWN	Reported DG	PSMD1	DOWN	Reported DG	co-exp
HDAC1	DOWN	Reported TF	STAT3	DOWN	Reported TF	co-exp
ACTR2	DOWN	Reported DG	ARPC5L	DOWN	Reported DG	co-exp
FGFR1	UP	Reported DG	CREBBP	UP	Reported TF	co-exp
HDAC1	DOWN	Reported TF	BHLHE41	UP	Reported DG	co-exp
NCOR1	DOWN	Reported TF	TRIM28	DOWN	Reported TF	co-exp
BCL6	UP	Reported DG	CTBP1	UP	Reported TF	co-exp
KAT2A	UP	Reported DG	KAT2A	UP	Reported DG	co-exp
STAT3	DOWN	Reported TF	DNMT1	UP	Reported TF	co-exp
STAT1	DOWN	Reported TF	STAT5A	DOWN	Reported TF	co-exp
HDAC1	DOWN	Reported TF	TRIM28	DOWN	Reported TF	co-exp

PGK1	DOWN	Reported DG	TPI1	DOWN	Reported DG	co-exp
HDAC1	DOWN	Reported TF	POU2F1	UP	Reported DG	co-exp
NCOR1	DOWN	Reported TF	CREBBP	UP	Reported TF	co-exp
ATP6V1C1	DOWN	Reported DG	STAT1	DOWN	Reported TF	co-exp
STAT1	DOWN	Reported TF	JMJD6	DOWN	Reported TF	co-exp
TOMM22	DOWN	Reported DG	TOMM70	DOWN	Reported DG	co-exp
JUN	DOWN	Reported TF	NCOR1	DOWN	Reported TF	co-exp
XIAP	DOWN	Reported DG	ACVR1B	DOWN	Reported DG	co-exp
CTBP1	UP	Reported TF	DNMT1	UP	Reported TF	co-exp
CREBBP	UP	Reported TF	KLF2	DOWN	Reported TF	co-exp
DNMT1	UP	Reported TF	E2F1	DOWN	Reported TF	co-exp
STAT3	DOWN	Reported TF	PGK1	DOWN	Reported DG	co-exp
UMPS	DOWN	Reported DG	GBE1	DOWN	Reported DG	co-exp
ATP6V1A	DOWN	Reported DG	RAB2A	DOWN	Reported DG	co-exp
ATP6V1A	DOWN	Reported DG	XIAP	DOWN	Reported DG	co-exp
SLC25A6	DOWN	Reported DG	TIMM10	DOWN	Reported DG	co-exp
ILF3	DOWN	Reported DG	TOMM22	DOWN	Reported DG	co-exp
NDUFA9	DOWN	Reported DG	NNT	DOWN	Reported DG	co-exp
CSNK1D	DOWN	Reported DG	PPM1B	UP	Reported DG	co-exp
ILF3	DOWN	Reported DG	RAB14	DOWN	Reported DG	co-exp
SLC25A6	DOWN	Reported DG	NNT	DOWN	Reported DG	co-exp
NDUFA9	DOWN	Reported DG	AFG3L2	DOWN	Reported DG	co-exp
NNT	DOWN	Reported DG	TIMM10	DOWN	Reported DG	co-exp
CD55	DOWN	Reported DG	MAN2A1	UP	Reported DG	co-exp
GSS	DOWN	Reported DG	GBE1	DOWN	Reported DG	co-exp

RAB14	DOWN	Reported DG	ATP6V1C1	DOWN	Reported DG	co-exp
ILF3	DOWN	Reported DG	RAB2A	DOWN	Reported DG	co-exp
NDUFA9	DOWN	Reported DG	ATP6V1C1	DOWN	Reported DG	co-exp
MTCH2	DOWN	Reported DG	TOMM22	DOWN	Reported DG	co-exp
ILF3	DOWN	Reported DG	MYH10	DOWN	Reported DG	co-exp
STAG2	UP	Reported DG	BZW2	DOWN	Reported DG	co-exp
ACTR2	DOWN	Reported DG	UBE2N	DOWN	Reported DG	co-exp
CRYAB	UP	Reported DG	CRYAB	UP	Reported DG	Direct interaction
STAT1	DOWN	Reported TF	STAT3	DOWN	Reported TF	Direct interaction
CTBP1	UP	Reported TF	ZEB1	UP	Reported TF	Direct interaction
MAF	DOWN	Reported TF	MAF	DOWN	Reported TF	Direct interaction
HDAC1	DOWN	Reported TF	DNMT1	UP	Reported TF	Direct interaction
STAT5A	DOWN	Reported TF	MAPK1	UP	Reported DG	Direct interaction
KAT2B	UP	Reported TF	CREBBP	UP	Reported TF	Direct interaction
STAT2	DOWN	Reported TF	STAT1	DOWN	Reported TF	Direct interaction
ZBTB16	UP	Reported DG	NCOR1	DOWN	Reported TF	Direct interaction
USF2	UP	Reported TF	USF2	UP	Reported TF	Direct interaction
BECN1	DOWN	Reported DG	BCL2	UP	Reported TF	Direct interaction
XIAP	DOWN	Reported DG	XIAP	DOWN	Reported DG	Direct interaction
BECN1	DOWN	Reported DG	BECN1	DOWN	Reported DG	Direct interaction
JUN	DOWN	Reported TF	MAPK1	UP	Reported DG	Direct interaction
HDAC1	DOWN	Reported TF	BCL6	UP	Reported DG	Direct interaction
MID1	UP	Reported DG	MID1	UP	Reported DG	Direct interaction
JUN	DOWN	Reported TF	STAT3	DOWN	Reported TF	Direct interaction
PAFAH1B1	DOWN	Reported DG	PAFAH1B1	DOWN	Reported DG	Direct interaction

DNMT1	UP	Reported TF	SIRT1	UP	Reported DG	Direct interaction
CTBP1	UP	Reported TF	KAT2B	UP	Reported TF	Direct interaction
SETDB1	DOWN	Reported TF	TRIM28	DOWN	Reported TF	Direct interaction
NCOR1	DOWN	Reported TF	BCL6	UP	Reported DG	Direct interaction
MAPK1	UP	Reported DG	BCL2	UP	Reported TF	Direct interaction
SIRT1	UP	Reported DG	JUN	DOWN	Reported TF	Direct interaction
BHLHE41	UP	Reported DG	BHLHE41	UP	Reported DG	Direct interaction
SIRT1	UP	Reported DG	BRINP1	UP	Reported DG	Direct interaction
TAF1	DOWN	Reported TF	JUN	DOWN	Reported TF	Direct interaction
E2F1	DOWN	Reported TF	SIRT1	UP	Reported DG	Direct interaction
BIN1	UP	Reported DG	BIN1	UP	Reported DG	Direct interaction
QKI	UP	Reported DG	RBFOX2	DOWN	Reported DG	Direct interaction
CTBP1	UP	Reported TF	CTBP1	UP	Reported TF	Direct interaction
BCL6	UP	Reported DG	BCL11A	UP	Reported TF	Direct interaction
NCOR1	DOWN	Reported TF	SIRT1	UP	Reported DG	Direct interaction
GSS	DOWN	Reported DG	GSS	DOWN	Reported DG	Direct interaction
FGFR1	UP	Reported DG	CREBBP	UP	Reported TF	Direct interaction
APLP2	DOWN	Reported DG	APLP2	DOWN	Reported DG	Direct interaction
STAT5A	DOWN	Reported TF	STAT5A	DOWN	Reported TF	Direct interaction
UBE2N	DOWN	Reported DG	MID1	UP	Reported DG	Direct interaction
SIRT1	UP	Reported DG	TRIM28	DOWN	Reported TF	Direct interaction
HDAC1	DOWN	Reported TF	BHLHE41	UP	Reported DG	Direct interaction
CREBBP	UP	Reported TF	CTBP1	UP	Reported TF	Direct interaction
CREBBP	UP	Reported TF	CREBBP	UP	Reported TF	Direct interaction
CREBBP	UP	Reported TF	CUX1	UP	Reported TF	Direct interaction

UBE2N	DOWN	Reported DG	ARIH1	DOWN	Reported DG	Direct interaction
TOR1A	DOWN	Reported DG	TOR1A	DOWN	Reported DG	Direct interaction
JUN	DOWN	Reported TF	BCL6	UP	Reported DG	Direct interaction
ZBTB16	UP	Reported DG	BCL6	UP	Reported DG	Direct interaction
JUN	DOWN	Reported TF	APLP2	DOWN	Reported DG	Direct interaction
SIRT1	UP	Reported DG	KAT2B	UP	Reported TF	Direct interaction
SRF	DOWN	Reported TF	NFYA	DOWN	Reported TF	Direct interaction
ZBTB7A	UP	Reported TF	BCL6	UP	Reported DG	Direct interaction
BCL6	UP	Reported DG	CTBP1	UP	Reported TF	Direct interaction
USF2	UP	Reported TF	MAF	DOWN	Reported TF	Direct interaction
KAT2A	UP	Reported DG	KAT2A	UP	Reported DG	Direct interaction
STAT1	DOWN	Reported TF	RXRA	DOWN	Reported TF	Direct interaction
STAT1	DOWN	Reported TF	E2F1	DOWN	Reported TF	Direct interaction
UMPS	DOWN	Reported DG	UMPS	DOWN	Reported DG	Direct interaction
SUN2	UP	Reported DG	SUN2	UP	Reported DG	Direct interaction
FLT1	UP	Reported variant/DG	SHC2	UP	Reported DG	Direct interaction
NCOR1	DOWN	Reported TF	TCF12	UP	Reported DG/TF	Direct interaction
ZBTB16	UP	Reported DG	ATP6AP2	DOWN	Reported DG	Direct interaction
JMJD6	DOWN	Reported TF	DIP2A	UP	Reported DG	Direct interaction
E2F1	DOWN	Reported TF	UCHL5	DOWN	Reported DG	Direct interaction
STAT3	DOWN	Reported TF	STAT6	DOWN	Reported TF	Direct interaction
UBE2N	DOWN	Reported DG	XIAP	DOWN	Reported DG	Direct interaction
CDKN1C	UP	Reported DG	CDKN1C	UP	Reported DG	Direct interaction
BCL11A	UP	Reported TF	SIRT1	UP	Reported DG	Direct interaction

TRIM28	DOWN	Reported TF	STAT3	DOWN	Reported TF	Direct interaction
DNMT1	UP	Reported TF	KAT2B	UP	Reported TF	Direct interaction
BIN1	UP	Reported DG	E2F1	DOWN	Reported TF	Direct interaction
KAT2B	UP	Reported TF	CUX1	UP	Reported TF	Direct interaction
NCOR1	DOWN	Reported TF	RXRA	DOWN	Reported TF	Direct interaction
CRYAB	UP	Reported DG	MDH2	DOWN	Reported DG	Direct interaction
CRY1	UP	Reported DG	CRY1	UP	Reported DG	Direct interaction
BECN1	DOWN	Reported DG	KAT2A	UP	Reported DG	Direct interaction
SRF	DOWN	Reported TF	RXRA	DOWN	Reported TF	Direct interaction
ACVR1B	DOWN	Reported DG	RXRA	DOWN	Reported TF	Direct interaction
WDR77	DOWN	Reported DG	QKI	UP	Reported DG	Direct interaction
LUC7L3	UP	Reported DG	LUC7L3	UP	Reported DG	Direct interaction
ALAS1	DOWN	Reported DG	ALAS1	DOWN	Reported DG	Direct interaction
TPI1	DOWN	Reported DG	SETDB1	DOWN	Reported TF	Direct interaction
CKAP4	DOWN	Reported DG	CKAP4	DOWN	Reported DG	Direct interaction
CRY1	UP	Reported DG	BHLHE41	UP	Reported DG	Direct interaction
ALAS1	DOWN	Reported DG	C2orf42	UP	Reported DG	Direct interaction
MOB4	DOWN	Reported DG	SETDB1	DOWN	Reported TF	Direct interaction
ITSN2	UP	Reported DG	FCHSD2	UP	Reported DG	Direct interaction
ANKRD28	UP	Reported DG	TGM2	UP	Reported DG	Direct interaction
TRAF3IP1	DOWN	Reported DG	OLFM1	DOWN	Reported DG	Direct interaction
ALAS1	DOWN	Reported DG	ZNF175	UP	Reported DG	Direct interaction
STAT5A	DOWN	Reported TF	TCF12	UP	Reported DG/TF	Direct interaction
BIN1	UP	Reported DG	CUX1	UP	Reported TF	Direct interaction
ITSN2	UP	Reported DG	ITSN2	UP	Reported DG	Direct interaction

ITSN2	UP	Reported DG	LUC7L3	UP	Reported DG	Direct interaction
TCF12	UP	Reported DG/TF	CREBBP	UP	Reported TF	Direct interaction
XIAP	DOWN	Reported DG	BCL2	UP	Reported TF	Direct interaction
TRIM28	DOWN	Reported TF	UBE2N	DOWN	Reported DG	Direct interaction
UCHL5	DOWN	Reported DG	TPI1	DOWN	Reported DG	Direct interaction
SIRT1	UP	Reported DG	BHLHE41	UP	Reported DG	Direct interaction
MYH10	DOWN	Reported DG	MYH10	DOWN	Reported DG	Direct interaction
SIRT1	UP	Reported DG	SIRT1	UP	Reported DG	Direct interaction
MAPK1	UP	Reported DG	CREBBP	UP	Reported TF	Direct interaction
CREBBP	UP	Reported TF	BCL6	UP	Reported DG	Direct interaction
ARIH1	DOWN	Reported DG	CRY1	UP	Reported DG	Direct interaction

Table S9: Potent drugs based on the gene expression profile (lacks selectivity) (red color denotes the selected target

Name	category	Targets	Score
HG-6-64-01	RAF inhibitor	ABL1, BRAF, CSF1R, EGFR, **FGFR1**, FLT3, KIT, MAPK11, PDGFRB, RET	97.04
BMS-536924	IGF-1 inhibitor	IGF1R, AKT1, CCNE1, CDK2, CYP3A4, ERBB2, INSR, KDR, LCK, **MAPK1**, MET, PDGFRA, PDGFRB	96.86
tivozanib	VEGFR inhibitor	**FLT1**, FLT4, KDR, KIT, PDGFRA, PDGFRB	96.12
LY-294002	MTOR inhibitor	MTOR, PIK3CD, PIK3CG, PIK3CA, PIK3CB, PLK1, PRKDC, AKT1, CHEK1, GSK3B, LCK, **MAPK1**, MAPK11, MAPK12, MAPK14, MAPK8, PDE2A, PRKCA, ROCK1, RPS6KB1, SGK1	95.91
SU-11652	Tyrosine kinase inhibitor	KDR, PDGFRB, CAMK1G, FGF2, **FGFR1**, **FLT1**, KIT, PDGFRA	94.46
ENMD-2076	FLT3 inhibitor	AURKA, FLT3, KDR, PDGFRA, SRC, CSF1R, EPHA1, **FGFR1**, FGFR2, FGFR3, FLT4, KIT, PTK2	91.65
regorafenib	FGFR inhibitor	ABL1, BRAF, DDR2, EPHA2, **FGFR1**, FGFR2, **FLT1**, FLT4, FRK, KDR, KIT, MAPK11, NTRK1, PDGFRA, PDGFRB, RAF1, RET, TEK	91.23
erdafitinib	FGFR inhibitor	**FGFR1,** FGFR2, FGFR3, FGFR4	90.78
nintedanib	FGFR inhibitor, PDGFR tyrosine kinase receptor inhibitor, VEGFR inhibitor	**FGFR1**, FGFR2, FGFR3, FGFR4, **FLT1**, FLT4, KDR, PDGFRA, PDGFRB	90.12
pazopanib	KIT inhibitor, PDGFR tyrosine kinase receptor inhibitor, VEGFR inhibitor	CSF1R, FGF1, **FGFR1**, FGFR3, **FLT1**, FLT4, ITK, KDR, KIT, PDGFRA, PDGFRB, SH2B3	89.78
ponatinib	Bcr-Abl kinase inhibitor, FLT3 inhibitor, PDGFR tyrosine kinase receptor inhibitor	ABL1, BCR, **FGFR1**, FGFR2, FGFR3, FGFR4, FLT3, KDR, KIT, LCK, LYN, PDGFRA, RET, SRC, TEK	89.27

regorafenib	FGFR inhibitor, KIT inhibitor, PDGFR tyrosine kinase receptor inhibitor, RAF inhibitor, RET tyrosine kinase inhibitor, VEGFR inhibitor	ABL1, BRAF, DDR2, EPHA2, **FGFR1**, FGFR2, **FLT1,** FLT4, FRK, KDR, KIT, MAPK11, NTRK1, PDGFRA, PDGFRB, RAF1, RET, TEK	89.12
sorafenib	FLT3 inhibitor, KIT inhibitor, PDGFR tyrosine kinase receptor inhibitor, RAF inhibitor, RET tyrosine kinase inhibitor, VEGFR inhibitor	BRAF, DDR2, **FGFR1, FLT1**, FLT3, FLT4, KDR, KIT, PDGFRB, RAF1, RET	88.19
sunitinib	FLT3 inhibitor, KIT inhibitor, PDGFR tyrosine kinase receptor inhibitor, RET tyrosine kinase inhibitor, VEGFR inhibitor	CSF1R, **FGFR1, FLT1**, FLT3, FLT4, KDR, KIT, PDGFRA, PDGFRB, RET	86.43
axitinib	PDGFR tyrosine kinase receptor inhibitor, VEGFR inhibitor	CSF1, **FLT1**, FLT4, KDR, PLK4	85.28
nintedanib	FGFR inhibitor, PDGFR tyrosine kinase receptor inhibitor, VEGFR inhibitor	**FGFR1**, FGFR2, FGFR3, FGFR4, **FLT1**, FLT4, KDR, PDGFRA, PDGFRB	85.06

Supplementary Figures

Exploring common therapeutic targets for neurodegenerative disorders using transcriptome study

S. Akila Parvathy Dharshini [1], Sherlyn Jemimah [1], Y.H Taguchi [2], M. Michael Gromiha [1*]

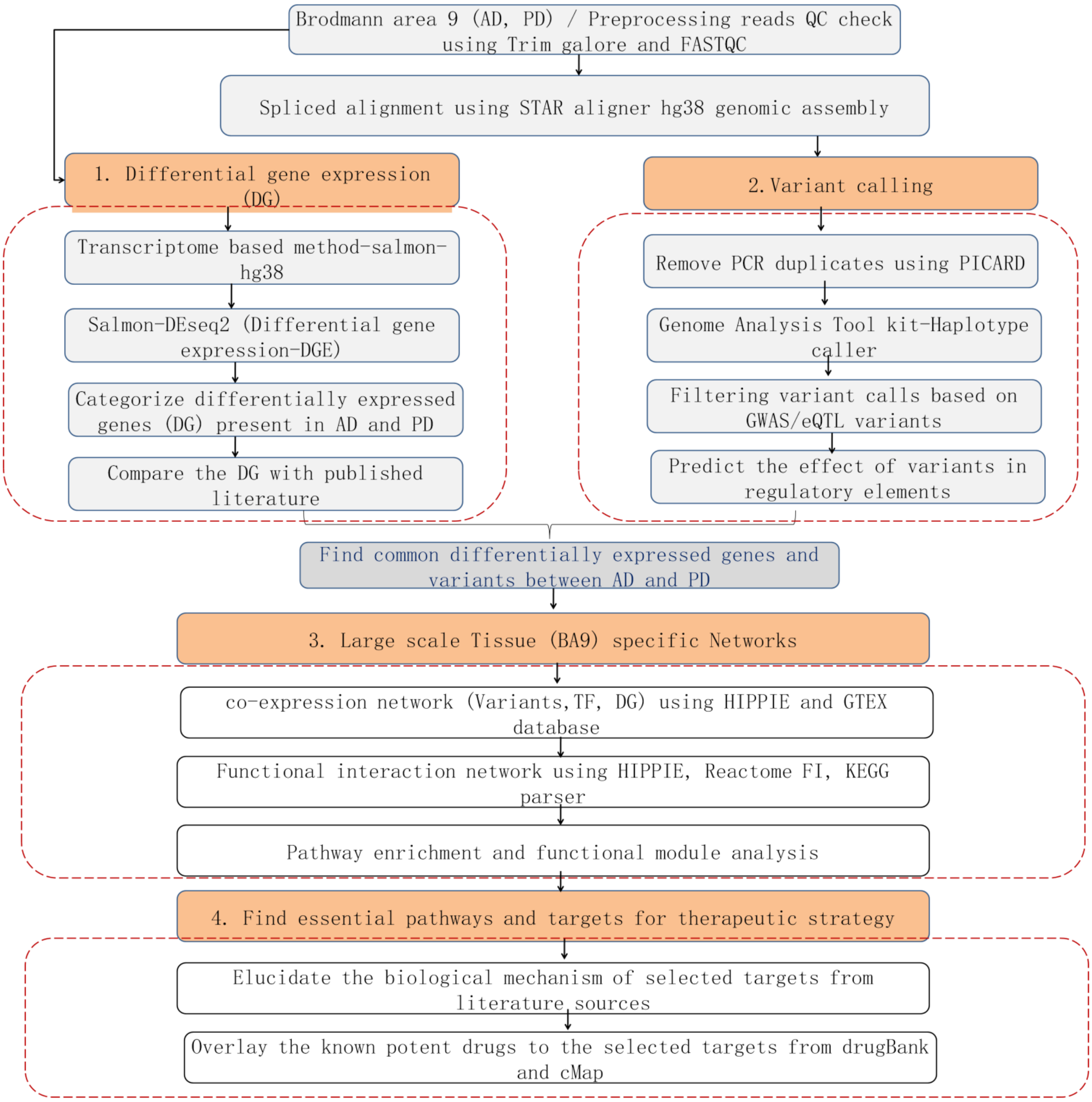

Figure S1: The workflow for variant calling, differential gene expression and large scale network analysis.

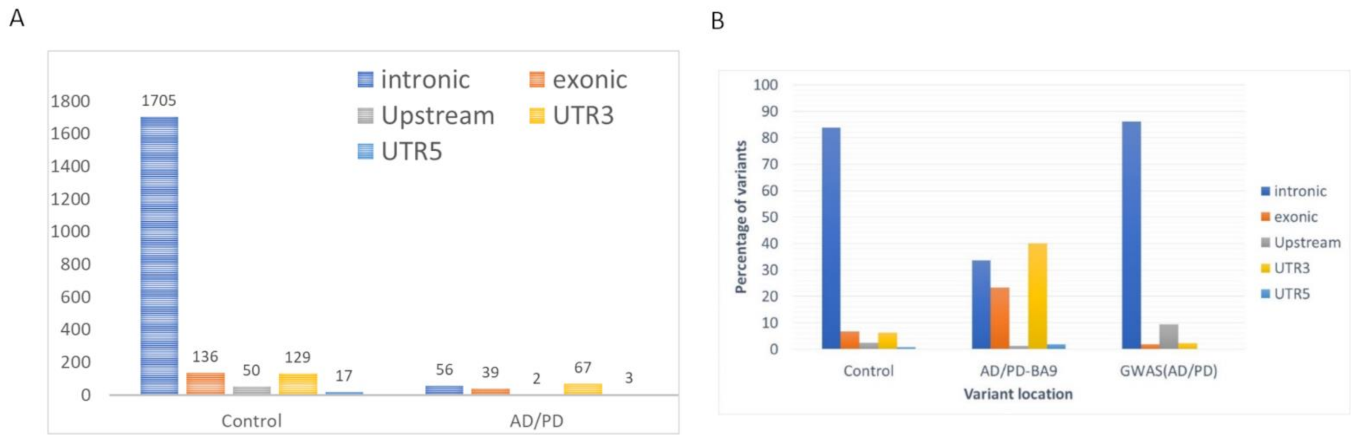

Figure S2: A] Genomic location of variants in control and disease samples. B] Percentage of variants located in various genomic locations

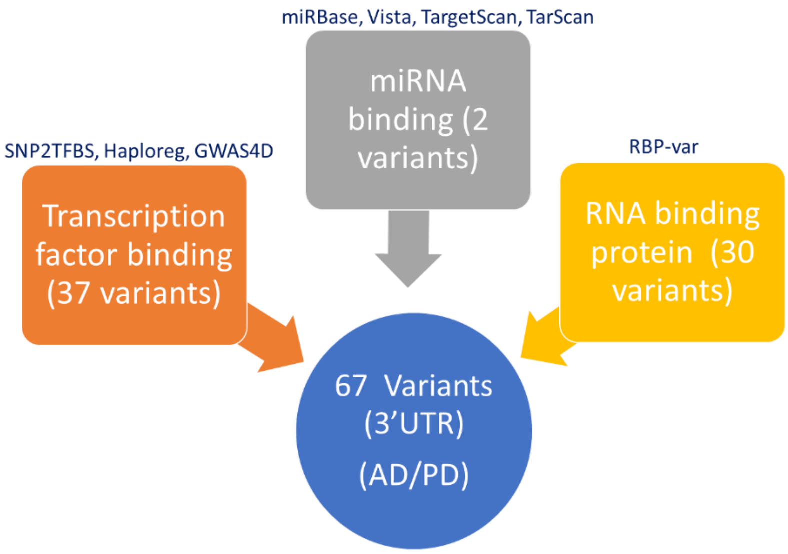

Figure S3: The effect of variants on TF, miRNA and RNA binding protein motifs

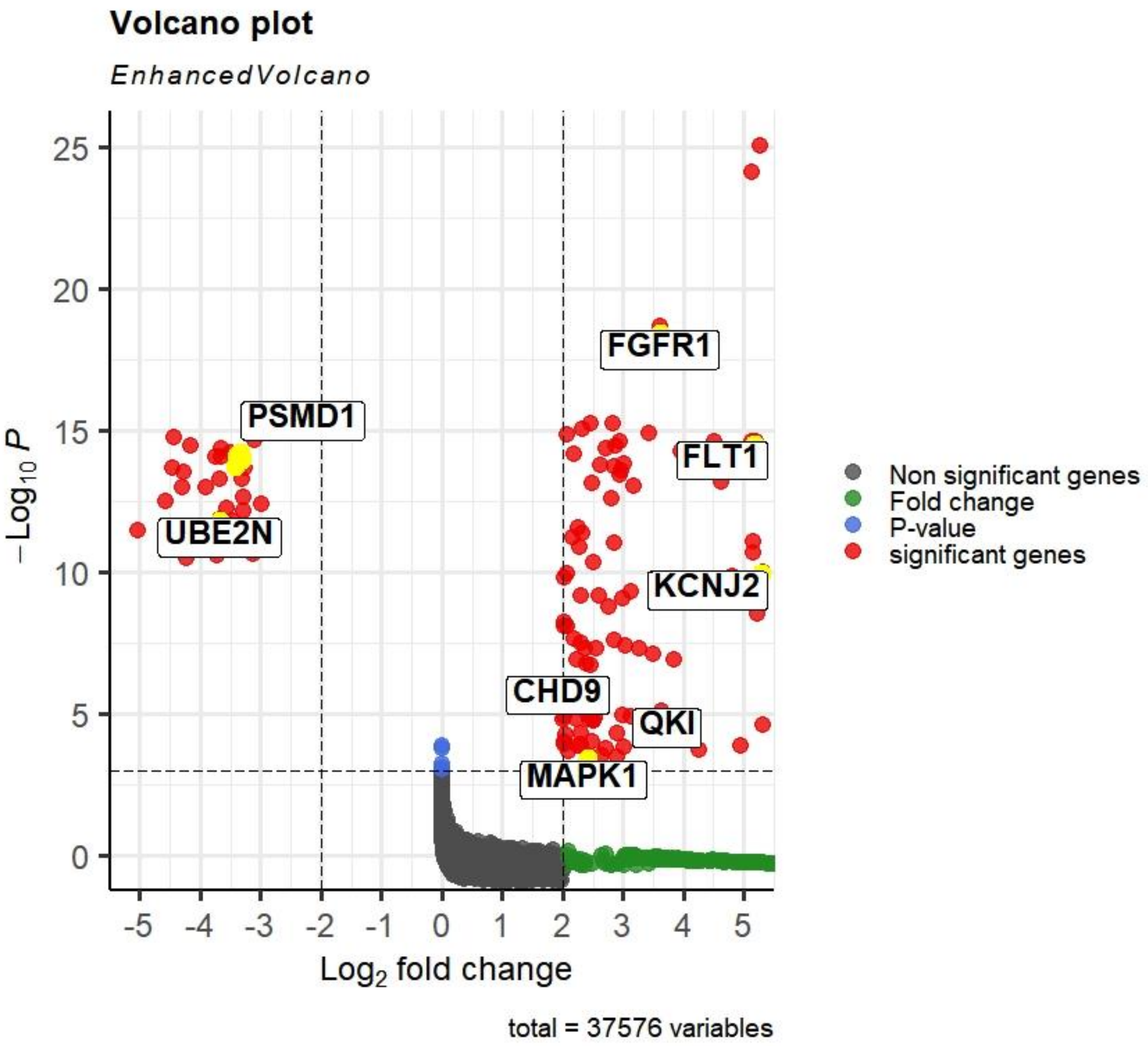

Figure S4: The volcano plot for differentially expressed genes

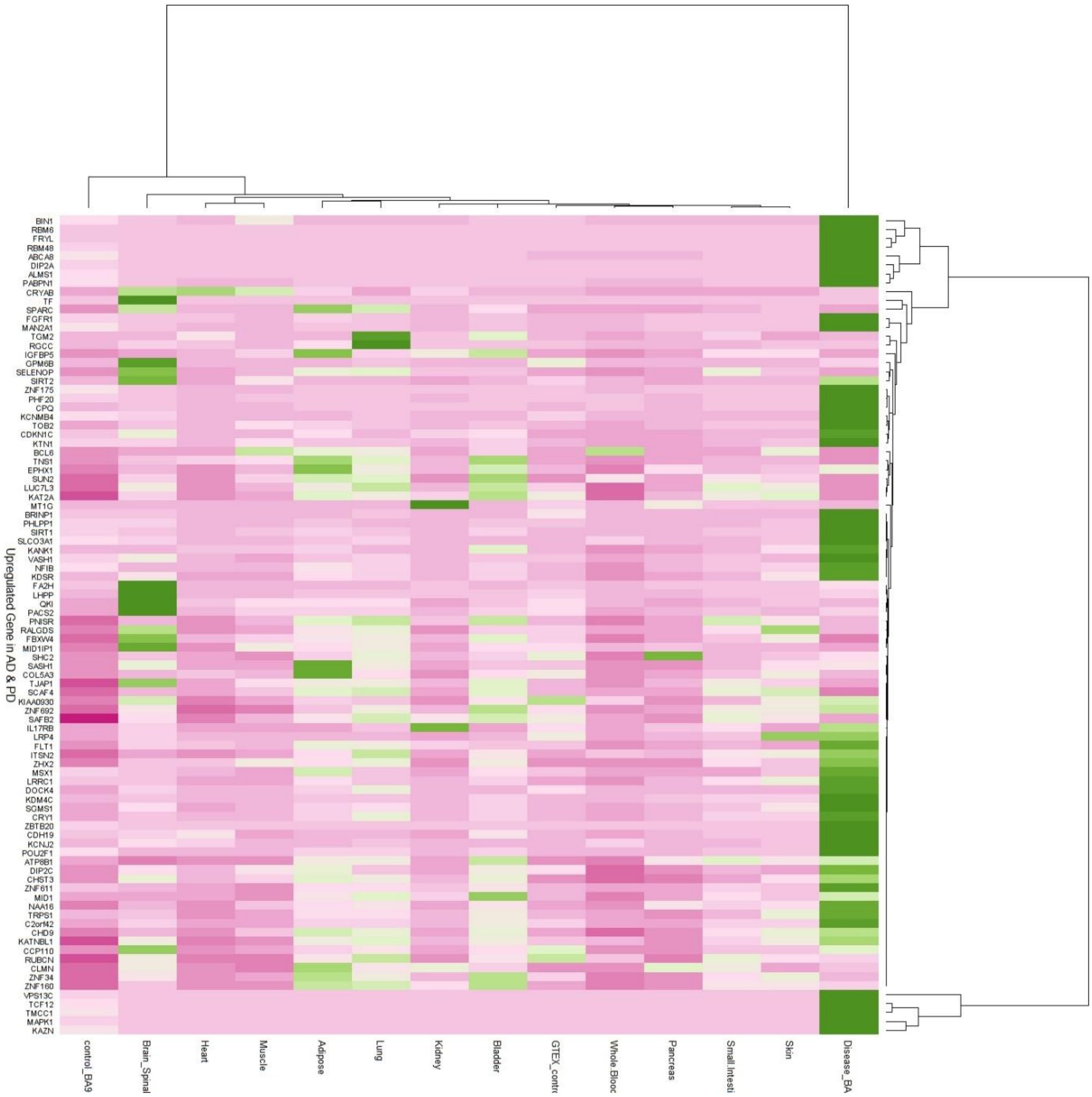

Figure S5: The expression profiles (TPM) of upregulated genes in AD & PD at various tissue locations

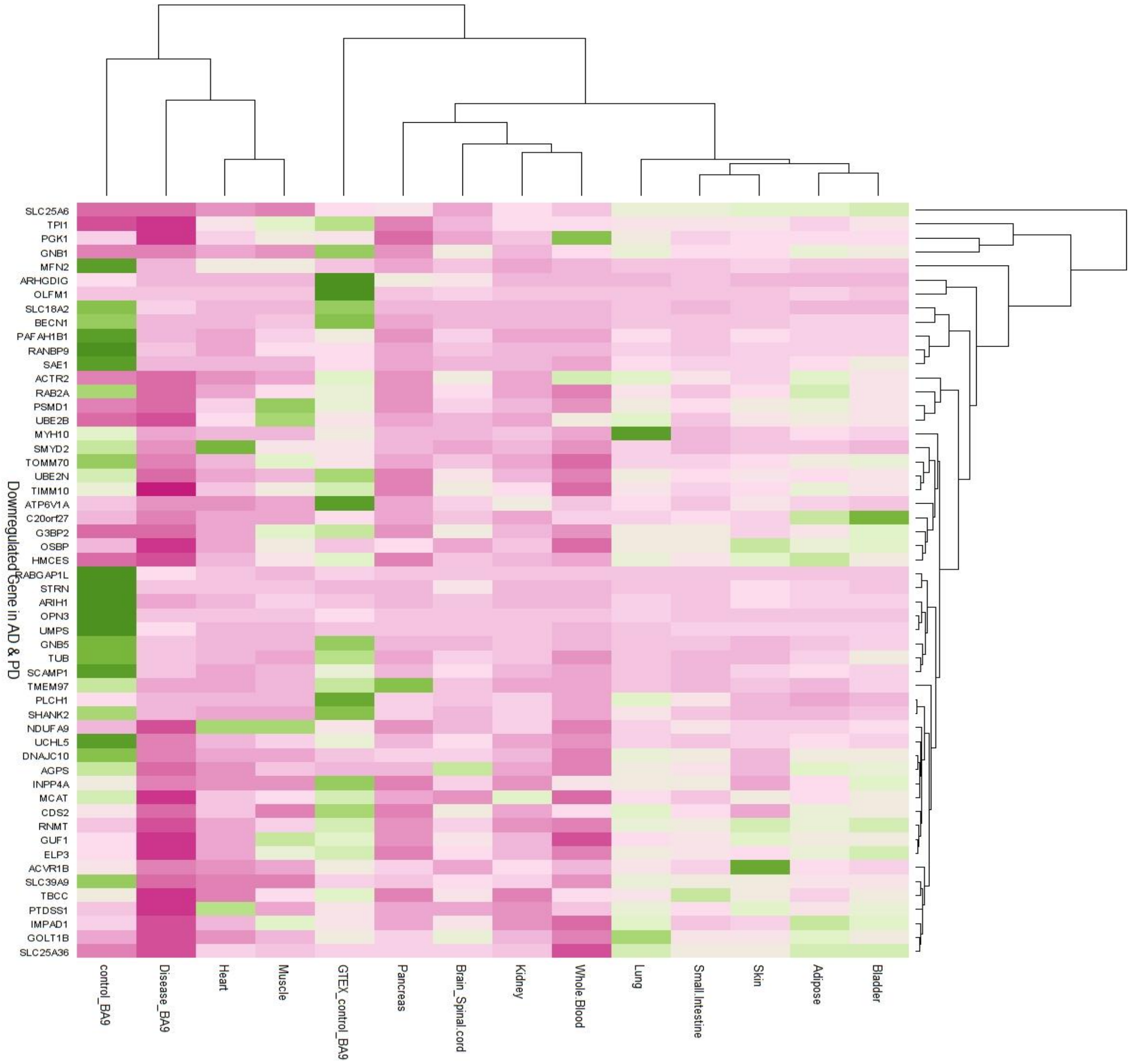

Figure S6: The expression profiles (TPM) of downregulated genes in AD & PD at various tissue locations

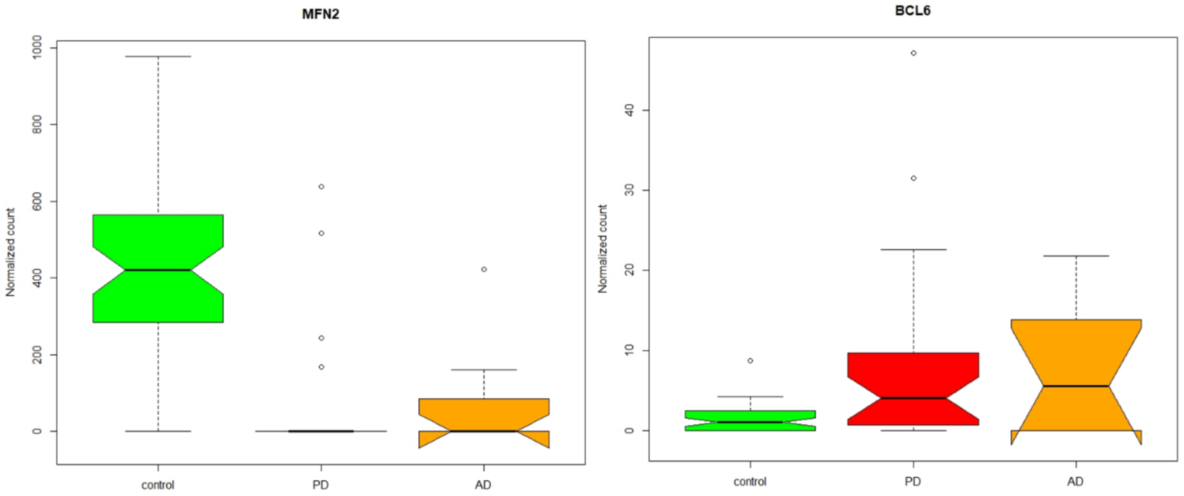

Figure S7: Normalized gene counts for MFN2 and BCL6 in control AD and PD disease samples

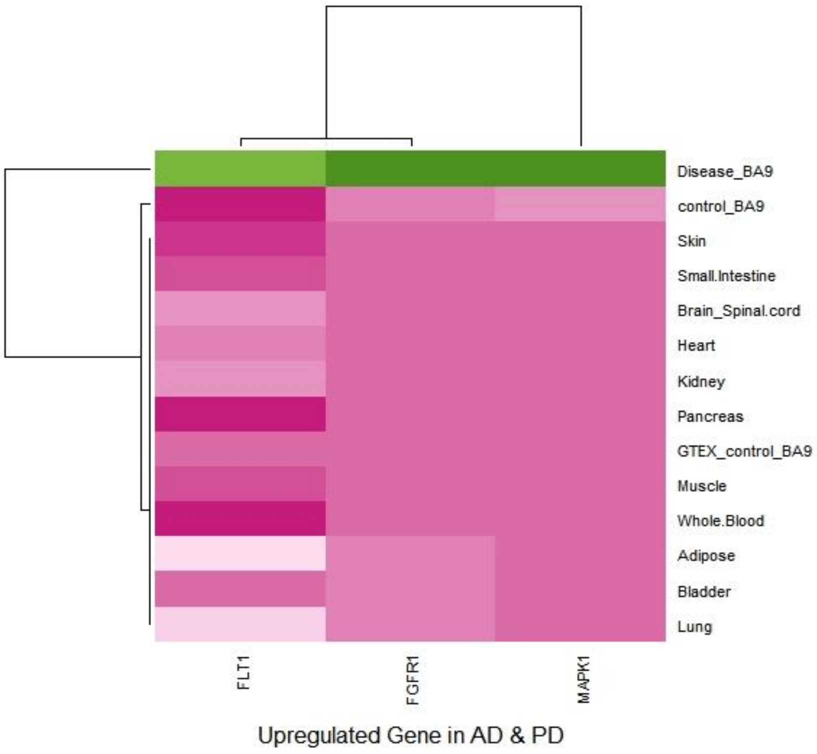

Figure S8: Expression profiles (TPM) of selected therapeutic targets at various tissue location
